# A wearable paper-based SGR/MCC microneedle array sensor for continuous glucose monitoring

**DOI:** 10.1038/s41378-026-01313-1

**Published:** 2026-06-03

**Authors:** Joseph Benjamin Holman, Talifhani Mushiana, Chen Yang, Yue Jin, Zhengdi Shi, Bensheng Qiu, Chengpan Li, Weiping Ding

**Affiliations:** 1 Department of Oncology, The First Affiliated Hospital of USTC, Division of Life Sciences and Medicine, and Department of Electronic Engineering and Information Science, University of Science and Technology of China, Hefei, China; 2 Hefei National Research Center for Physical Sciences at the Microscale, and Department of Chemistry, University of Science and Technology of China, Hefei, China; 3 Medical Imaging Center, School of Information Science and Technology, University of Science and Technology of China, Hefei, China

**Keywords:** Electronic devices, Biosensors

## Abstract

Continuous glucose monitoring (CGM) is a promising approach for managing blood glucose levels in individuals with diabetes. However, the emerging microneedle (MN) technologies for colorimetric or electrochemical detection of glucose in sampled dermal interstitial fluid (ISF) remain far from practical CGM due to many concerns. Herein, we propose a new disposable solid MN array on a flexible paper substrate, integrated with reusable electronics and a mobile application, for electrochemical glucose sensing in ISF. The paper substrate was prepared by infusing paper with UV-cured flexible gingiva mask resin, ensuring body comfort. The solid MN array was fabricated from a newly developed composite of UV-cured biocompatible surgical guide resin (SGR) and microcrystalline cellulose (MCC), using a simple self-developed fabrication technique, ensuring safe skin interaction. The paper-based composite MN arrays were converted into conductive biosensing electrodes by coating them with graphene ink and additional functional modifiers via airbrushing, and subsequently integrated into a wearable patch for continuous ISF glucose monitoring. The resulting MN array electrodes exhibited excellent analytical performance in monitoring glucose of ex vivo porcine plasma, hydrogel-based artificial skin, and in vivo mice, with high sensitivity, selectivity against interferents, and robust stability. This study provides a simple, cost-effective, and eco-friendly path for the development of wearable electroanalytical devices toward practical CGM.

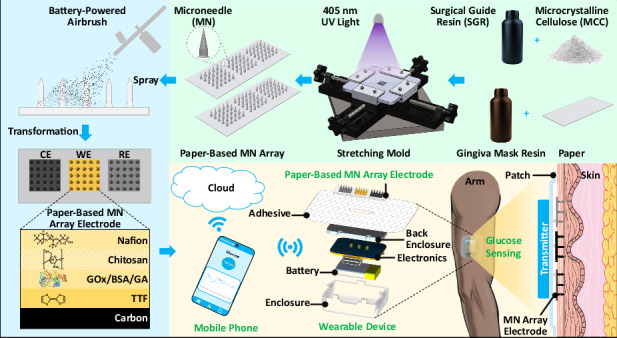

## Introduction

With the emergence of diverse new technologies, sensors for continuous glucose monitoring (CGM) have undergone significant development^1,2^. Among these, transdermal wearable sensors have attracted considerable interest due to their minimally invasive nature, leading to the development, demonstration, and commercialization of various CGM devices^3,4^. However, limited patient adherence and acceptance are still a significant hurdle, primarily resulting from inherent invasiveness (though minimal) and the high cost of current CGM systems^5^. Conventional CGM devices typically employ a flexible sensor strip, 5–11 mm in length, which is inserted subcutaneously into the hypodermis^6^. This insertion process can cause discomfort for the wearer, and the insertion site of the sensor itself presents physiological limitations. The hypodermis, compared to the dermis, has a less dense capillary network, which may contribute to discrepancies between glucose concentrations in interstitial fluid (ISF) and those in the bloodstream^7,8^.

Currently, microneedle (MN) array sensors represent a promising alternative for CGM^9,10^, facilitating painless insertion and accessing only the dermis. MN array sensors for CGM can be classified into hollow and solid types. Hollow MN arrays have a built-in conduit for extracting ISF from the skin through capillary action or suction^11–13^, with an electrode fabricated externally on the substrate to analyze the collected ISF. However, hollow and porous MN arrays are prone to clogging and exhibit a lag in glucose measurements due to the time required for ISF extraction^14^. Although this can be improved by filling the conduit with conductive and electroactive materials^6^, the effective electrode area is limited. To address these limitations, recent studies have explored solid MNs with conductive coatings^11^.

For solid MN arrays used in CGM, materials such as silicon^15^, poly(methyl methacrylate)^9^, resin^16,17^, poly(glycidyl methacrylate)^18^, hydrogel^19^, and polystyrene^20^ have been adopted for the MN body. These arrays are fabricated using techniques such as molding, micromachining, photolithography, drawing lithography, and printing^21–24^. To function as direct electrodes, most of these MN bodies require coating with precious metals such as gold^15,16,25^, platinum^9^, and silver^15,16,18^. However, these metallization processes are expensive and require specialized equipment. Recently, graphene inks have been explored as conductive coatings, demonstrating the potential to reduce both the complexity and cost of converting non-conductive MN arrays into conductive MN arrays (i.e., electrodes)^20,26^. Regarding the substrate for solid MN arrays, the above-mentioned MN materials are also used. While the rigidity of the MN body is essential for skin penetration, rigid substrates compromise wearer comfort due to their lack of flexibility^27^. In addition, these materials are usually non-breathable and non-biodegradable, which may cause skin irritation and pose environmental concerns^28^.

Paper has proven to be promising as an alternative substrate for wearable devices because it is inexpensive, flexible, breathable, and biodegradable. The use of paper also enhances the disposability of wearable devices, thereby reducing the risk of infection. To date, various paper-based wearable sensors have been developed, typically targeting readily accessible biofluids such as sweat^28,29^. Some studies have explored the fabrication of hollow or porous MN arrays on paper substrates for transdermal access to ISF and subsequent colorimetric analysis^30–34^. However, integrating these MN arrays on paper for continuous monitoring still presents limitations, such as the pressure gradient requirement in sampling with hollow MN arrays and the inherently limited continuous sampling capability of porous MN arrays.

**Fig. 1:**
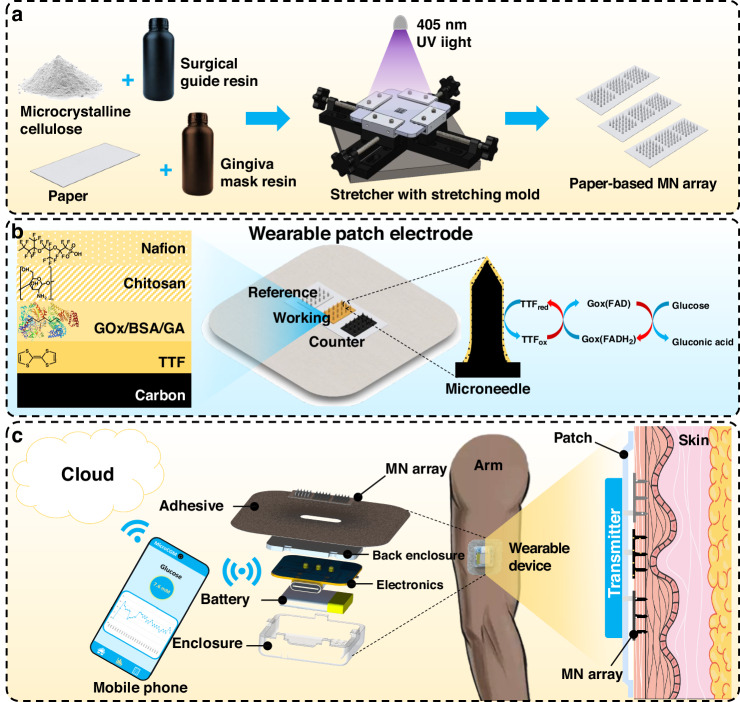
Schematic diagram of the disposable paper-based MN array patch for wireless CGM. **a** Fabrication of the paper-based MN array. **b** MN array electrode fabrication and modification. **c** Wearable MN array patch for CGM

In this article, we present a disposable wearable paper-based solid SGR/MCC MN array for the electrochemical monitoring of glucose in ISF. The solid MN arrays were fabricated from a new composite of UV-curable biocompatible surgical guide resin (SGR) and microcrystalline cellulose (MCC) to ensure the safety of MNs when interfacing with the skin. The substrate was made of paper infused with UV-curable flexible gingiva mask resin. First, the MN array was molded using a stretchable mold with the aid of a custom-built bi-directional stretcher, which enabled the casting of the viscous MN composite (Fig. 1a). The MN array was then coated with a graphene ink to render it conductive and sequentially modified with tetrathiafulvalene (TTF), glucose oxidase, chitosan, and Nafion using an airbrush for glucose sensing (Fig. 1b). Finally, the functionalized MN array was integrated into an adhesive patch and paired with reusable electronics and a mobile application to enable CGM (Fig. 1c). The wearable paper-based MN array demonstrated good analytical performance (characterized by sensitivity and selectivity against potential interferents) and robust stability during extended use. Moreover, the sensor’s potential for CGM was validated through testing with ex vivo porcine plasma, hydrogel-based artificial skin, and in vivo mice. This study provides a simple and inexpensive method for fabricating solid MN arrays on a paper substrate and offers a new platform for CGM.

## Materials and methods

### Materials

Biocompatible dental 3D printing resins (iF3165 and iF3161) were purchased from Guangzhou iFUN Technology Co., Ltd. (China). Potassium ferricyanide, dopamine (DA), uric acid (UA), tetrathiafulvalene (TTF), glutaraldehyde (GA), glucose oxidase (GOx) (>180 U/mg from *Aspergillus niger*), and Nafion were obtained from Shanghai Macklin Biochemical Technology Co., Ltd. (China). Acetone, ethanol, sodium chloride, potassium chloride, and ascorbic acid (AA) were purchased from Sinopharm Chemical Reagent Co., Ltd. (China). Phosphate buffer saline (PBS) powder was obtained from Biosharp Life Sciences (China). Graphene conductive ink (LN-GCI-3) was supplied by Leadernano Technology Co., Ltd. (China). Ag/AgCl ink (JLL10) & R2 were purchased from Ju Long Hui Na (Shanghai, China). Waxfilm was obtained from Biofount Vande (Beijing) Biotechnology Co., Ltd. Porcine plasma (aseptically filtrated red blood cells) was purchased from Pingrui Biotechnology Co., Ltd. (Zhengzhou, China). Endothelial cell complete medium (ECM) was obtained from ScienCell Research Laboratories (California, USA). Bovine serum albumin (BSA) and Calcein-AM were purchased from Aladdin Industrial Corporation (Shanghai, China). D-(+)-Glucose and Propidium iodide (PI) were obtained from Sangon Biotech Co., Ltd. (Shanghai, China).

### Fabrication of the MN array

The master mold of the MN array (5 × 5) was designed in SolidWorks and fabricated using a high-precision 3D printer (S130, BMF Material Technology Inc.) at the Engineering and Materials Experiment Center of the University of Science and Technology of China (USTC), Anhui, China. AB silicone rubber was used to create a negative, stretchable mold of the MN array. This AB silicone rubber mold was then used to replicate the MN array, using the UV-curable, biocompatible SGR/MCC composite with a backing layer made of paper soaked in a biocompatible, flexible 3D printing resin. During fabrication, the rubber mold was stretched using a custom bi-directional stretcher built from commercially available parts except for the 3D-printed grip pads, which were custom-designed (Supplementary Table S1), following a process similar to that previously described in ref. ^35^. The reusable master mold and AB silicon rubber mold minimize the need for repeated 3D printing when producing large quantities of MN arrays.

### Mechanical properties testing of the MN array

A SENS C22-102 universal material testing machine was utilized to investigate the mechanical properties of the composite and the strength of the MN array through compression testing. The MN array was placed on the lower plate, with an initial distance of 2.00 mm separating the upper plate and the tips of the MNs. The upper plate was moved towards the MNs at a constant speed of 1 mm/min. Load and displacement data were recorded at intervals of 0.01 s to generate the load-displacement curve.

### Skin insertion of the MN array

To evaluate the skin insertion ability of the MN array, both simulated skin and mouse skin were used. The simulated skin was prepared using six layers of Parafilm M, as previously described in the literature, to mimic the structural properties of human skin^36^. The MN array was applied to the six-layer Parafilm model and manually pressed for 30 s. After insertion, the array was removed, and each layer of the model was examined under a microscope to assess penetration depth. Insertion efficiency was calculated using Eq. (1):

1 $${\boldsymbol{E}}\left( \% \right)=\frac{{\boldsymbol{H}}}{{{\boldsymbol{T}}}_{{\boldsymbol{mn}}}}\times {\bf{100}} \%$$

where ***E*** represents the insertion efficiency, ***H*** is the number of holes observed in the waxfilm layer, and ${{\boldsymbol{T}}}_{{\boldsymbol{mn}}}$ is the total number of microneedles in the array.

For the mouse skin insertion experiments, abdominal skin excised from male BALB/c mice was used. The hair on the mouse’s abdomen was shaved, and the skin was excised, stretched, and secured onto a block of polystyrene foam. The MN array was applied to the skin and manually pressed with the thumb for 30 s. After removal of the MN array, the skin was fixed in tissue fixative solution, followed by hematoxylin and eosin (HE) staining to visualize and assess microneedle penetration. All animal experiments in this study were approved by the Animal Ethics Committee of the USTC (approval number: USTCACUC28120125129).

### Development of the MN array electrodes

For glucose sensing, the MN array was modified with various materials to develop electrochemical sensing electrodes, namely, reference electrode (RE), counter electrode (CE), and working electrode (WE). To create the conductive layer, vinyl sheet stickers (0.15 mm in thickness) with 5 × 5 mm cutouts were used as stencils. The RE and conductive tracks were fabricated by spraying Ag/AgCl ink onto the MN through a stencil. The CE and WE were fabricated by spraying an optimized carbon ink formulation (5 g of graphene conductive ink and 20 g of acetone, maintaining a 4:1 solvent-to-ink ratio) for ~10 s, with the airbrush nozzle held ~5 cm from the MN array. The WE was further modified by sequentially deposition of TTF solution (10 mM; prepared in ethanol), a mixture of GA (3%), GO× (20 mg/mL), and BSA (20 mg/mL) at a volume ratio of 1:1:1, followed by chitosan solution (0.5%) and Nafion solution (10%; prepared in ethanol). The conductive tracks connecting the electrodes were insulated by covering them with a ply of tissue paper impregnated with gingiva dental resin and curing it in place under UV light. After curing, the substrate was carefully trimmed along the conductive tracks using scissors. The fabricated MN array electrodes were dried and stored at 4 °C.

### Morphological analysis of the MN array electrodes

The MN array electrodes were coated with gold using a vacuum sputter (Leica EM ACE200 coating system, Leica Microsystems). The microstructure of the electrodes was evaluated using scanning electron microscopy (SEM), and the elemental composition was verified using energy-dispersive X-ray spectroscopy (EDS) (ZEISS EVO 18, Carl Zeiss, Germany) at the Engineering and Materials Experiment Center in USTC. The chemical composition of the MN array electrodes was confirmed by X-ray photoelectron spectroscopy (XPS).

### Electrochemical characterization of the MN array electrodes

Characterization of the MN array electrodes was performed at room temperature using a CHI660D potentiostat (CH Instruments, China). Three primary electrochemical techniques were employed: cyclic voltammetry (CV), amperometry, and electrochemical impedance spectroscopy (EIS). For the working electrode characterization, the WE lead from the potentiostat was connected to the WE of the MN array electrodes, while RE and CE were connected to the corresponding leads on the potentiostat. Amperometric measurements were conducted by applying a constant voltage of 0.3 V (versus the Ag/AgCl reference) to the WE. CV measurements were performed over a voltage range of −0.2 to 0.6 V. EIS was carried out for each functional layer of the sensor probe to investigate its electrical characteristics, using 0.1 M PBS as the electrolyte and a frequency range of 10 Hz–10 kHz. The output currents and impedances from the MN array sensor were recorded for all electrochemical measurements and used to plot current responses and construct calibration curves.

### Glucose-sensing performance testing

The glucose-sensing performance of the MN array electrodes was evaluated in various simulated samples, including PBS containing glucose, glucose-spiked porcine plasma, and a chitosan-agarose hydrogel. The hydrogel was prepared by mixing 1.5% agarose and 0.5% (w/v) chitosan in 1% (v/v) acetic acid solution^37^. First, MN array electrodes were inserted into a waxfilm to cover the base of the MN. Then, the arrays were immersed in the above-simulated samples, and the current response was recorded to evaluate glucose-sensing performance.

The selectivity of the MN array electrodes was tested against potential interferences from various chemical species and electroactive molecules. The MN array electrodes were immersed in PBS containing NaCl (1 mM), KCl (1 mM), dopamine (0.1 mM), ascorbic acid (0.1 mM), and uric acid (0.1 mM), and the current response was recorded and analyzed. To assess repeatability, three independently fabricated MN array electrodes (i.e., MN array Electrode A, B, and C) were used to measure glucose in the same solution.

### Glucose-sensing performance analysis

The sensing performance was evaluated by calculating two key parameters: sensitivity and limit of detection (LoD). The sensitivity of the MN array electrodes was determined by calculating the slope of the calibration curve. The mean current response (A) was plotted against the corresponding glucose concentration (mM). The sensitivity was expressed as the slope of the linear regression line (A/mM). The LoD was calculated using the method described by ref. ^38^. First, the Limit of Blank (LoB) was determined using Eq. (2):

2 $${\rm{LoB}}={\rm{mean\_blank}}+1.645({\rm{SD\_blank}})$$

where mean_blank is the mean current response of the blank sample (0 mM glucose) and SD_blank is its standard deviation.

The LoD was then calculated using Eq. (3):

3 $${\rm{LoD}}={\rm{LoB}}+1.645({\rm{SD\_low\; concentration\; sample}})$$

where SD_low concentration sample is the standard deviation of the lowest non-zero glucose concentration measured. The LoD in current units (A) was converted to concentration units (mM) using the calculated sensitivity.

### Cytotoxicity assay

The MN array was inserted into the ECM covered with a parafilm layer to mimic a skin-like environment and incubated for 24 h. The resulting medium was then used to culture primary human umbilical vein endothelial cells (HUVECs) stored in our lab. Here, HUVECs at passages 8–10 times were used. First, HUVECs were suspended in ECM, and 100 μL of the cell suspension was seeded into a 96-well plate (5000 cells per well). After 24 h of incubation, the cells were stained with Calcein-AM (2 μM) and PI (10 μg/mL) dyes at 37 °C for approximately 20 min. Finally, fluorescence images were captured using an inverted fluorescence microscope (IX73; Olympus Corporation, Tokyo, Japan), and cell viability was quantified using ImageJ software.

### In vivo glucose monitoring

Male BALB/c mice were used to assess the biocompatibility of the MN array electrodes in a proof-of-concept CGM. The mice were housed in individually ventilated enclosures and provided with standard laboratory food and water when they were not undergoing experiments. Before the glucose monitoring, mice were fasted for 5 h. The mice were anesthetized using an isoflurane vaporizer, and the MN array electrodes were attached to the shaved and cleaned skin. After a 15-min stabilization period, ISF glucose levels were monitored using the MN array electrodes at 15-min intervals. Meanwhile, blood samples were collected from the tail every 30 min (to minimize stress on the mice). Blood glucose levels were measured as a reference using a commercially available glucose meter. After glucose monitoring, the mice were euthanized, and the tissues of major organs were harvested for HE staining and histological analysis.

## Results

### Fabrication and characterization of the MN array

**Fig. 2:**
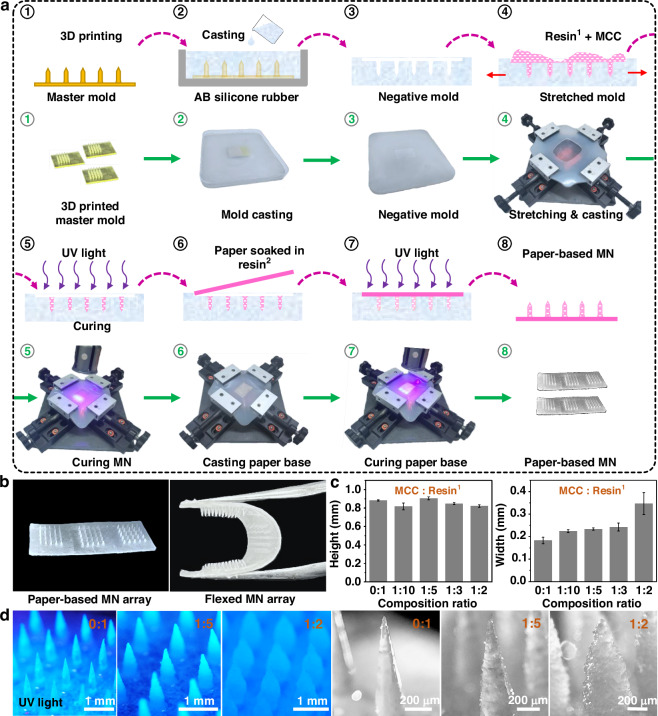
Fabrication and characterization of the paper-based MN array. **a** Schematic of the MN array fabrication. **b** Images of the paper-based MN array and its flexibility. **c** Height and width of MNs at various weight ratios of MCC: SGR (resin^1^). **d** Close-up images of the MN array (under UV Light) and a single MN under various composition ratios

The MN array was fabricated using a double-casting technique with a stretchable mold (Fig. 2a). To enable controlled stretching of the mold during casting, a custom bi-directional stretcher was developed, as shown in Supplementary Fig. S1. This device allows the flow of the viscous resin composite into the mold cavity, ensuring the formation of a well-defined MN array (Supplementary Fig. S2). Each MN in the array has a cylindrical body with a conical tip, a base diameter of 300 μm, and a height of 1 mm. The center-to-center spacing between adjacent MNs is 1000 μm. This geometry was designed to produce sharp tips capable of easily piercing the skin (Supplementary Fig. S3). The fabrication process began with 3D printing the master mold, which was then cleaned in an ultrasonic bath with isopropyl alcohol (➀). AB silicone rubber (part A: part B = 1:1) was poured over the master mold (➁) and cured at room temperature for 24 h to create a stretchable negative mold (➂). The SGR (resin^1^) and MCC composite was applied to the stretched AB silicon rubber negative mold, after which the mold was released from stretching, allowing excess resin to accumulate on top (➃). The excess resin was removed from the surface of the negative mold to ensure that the hard resin is confined to the needle geometry. The composite was then cured under 405 nm UV light (➄). After curing, a flexible backing layer was formed as a substrate by casting a piece of paper soaked in flexible dental resin (resin^2^) onto the surface of the negative mold (➅) and cured under UV light for 10 s (➆). Finally, the paper-based MN array was peeled off the negative mold for use (➇). Both the MN array fabricated with a new composite (the mixture of MCC and resin^1^) as the MN structure and the flexible paper substrate achieve a balance between strength and flexibility for transdermal applications (Fig. 2b). The composition ratio (i.e., MCC: Resin^1^) had a minimal effect on the height of the MNs but significantly affected the width of the MNs (Fig. 2c). MNs fabricated without MCC (0:1) displayed significant shrinkage, while MNs produced with high amounts of MCC (1:2) exhibited deformities (Fig. 2d). To determine the optimal composition ratio for the MNs, uniaxial tension testing of the polymer (Supplementary Fig. S4), finite element analysis (Supplementary Fig. S5), and compression testing (Supplementary Fig. S6) were performed. Based on the force per needle versus displacement curve, the failure thresholds were observed at 1 N/needle, 1.5 N/needle, and 3 N/needle for MCC: SGR (resin^1^) weight ratios of 0:1, 1:5, and 1:2, respectively (the observation also indirectly confirmed the strong adhesion between the microneedles and the paper substrate with resin^1^, as failure forces surpassed insertion and wear forces^39^, demonstrating low detachment risk). In addition, the insertion ability and efficiency of the fabricated MN arrays were also evaluated on a parafilm skin model (Supplementary Fig. S7). Considering the physical, mechanical, and insertion characteristics of the MN array, the w/w ratio of 1:5 was selected for subsequent experiments.

### Fabrication and characterization of the MN array electrodes

**Fig. 3:**
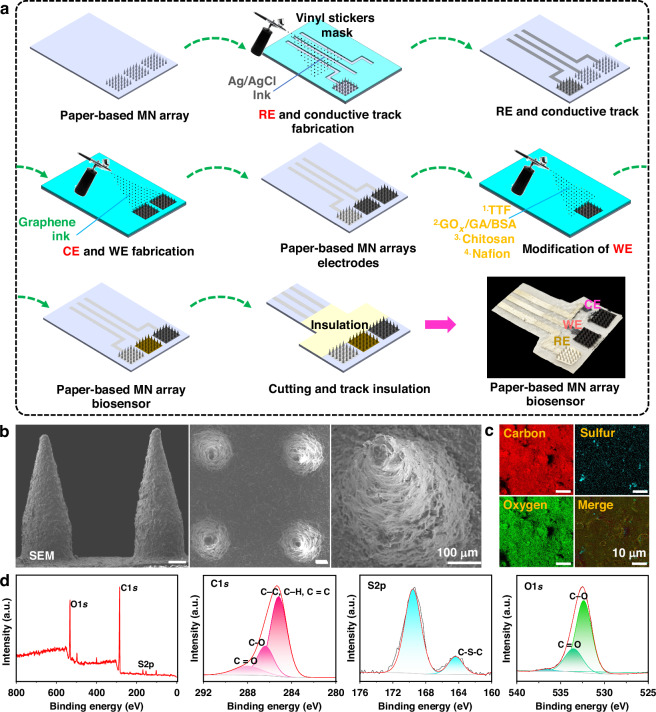
Transformation of the paper-based MN arrays into conductive carbon electrodes. **a** Schematic diagram of the deposition of materials on the paper-based MN arrays. **b** SEM images of the modified MNs as the WE. **c** EDS elemental mapping of the modified MNs, showing the presence of carbon, sulfur, and oxygen. **d** XPS measurements of the WE, showing survey peaks for C1s, S2p, and O1s

Once the fabrication of the paper-based MN array was complete, the MN array was converted into conductive electrodes and functionalized as a biosensing electrode using an airbrush (Fig. 3a). First, the RE and conductive tracks for connecting the electrodes to the potentiostat were deposited on the paper-based MN array using Ag/AgCl ink. The WE and CE were then formed via spray deposition of graphene-based conductive ink. The resin-infused paper and the rapid evaporation of the acetone ensured a uniform surface coating and significantly reduced the diffusion of the graphene ink into the paper. A TTF mediator layer was subsequently applied to the WE via spray coating. Due to its insolubility in water, TTF is stable when immobilized in aqueous environments, making it a promising mediator in transdermal systems^26^. Next, a mixture of GOx, GA (crosslinker), and BSA (stabilizer) was sprayed onto the TTF-modified WE to form the biosensing layer. Finally, the biosensing layer was protected to enhance its stability by coating with Chitosan and Nafion membranes. Figure 3b shows SEM images of the WE from multiple perspectives, while Fig. 3c confirms the presence and the uniform distribution of carbon, sulfur, and oxygen on the WE surface using EDX mapping analysis. To further clarify the specific surface composition, XPS measurements were performed (Fig. 3d). The peaks confirmed the presence of carbon and sulfur on the WE surface. Collectively, these results demonstrated the successful transformation of the paper-based MN arrays into MN array electrodes.

### Electrochemical characterization of the WE

**Fig. 4:**
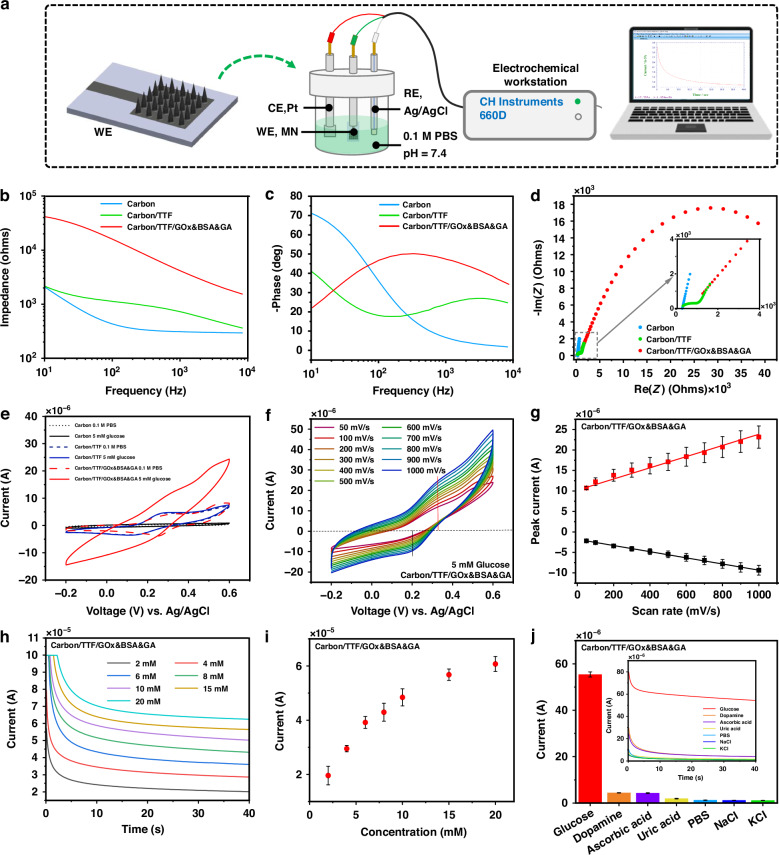
Characterization of the working electrode. **a** Schematic of the WE characterization setup. Subgraphs (**b**) and (**c**) show impedance and phase under different frequencies, respectively, for the carbon, carbon/TTF, and carbon/TTF/GOx&BSA&GA MN array electrodes in 0.01 M PBS. **d** Nyquist plot for the carbon, carbon/TTF, and Carbon/TTF/GOx&BSA&GA MN array electrodes. **e** Cyclic voltammetry of the WE in 0.01 M PBS and 5 mM glucose. **f** Cyclic voltammetry of the WE in a 10 mM glucose solution at different scan rates. **g** Calibration curves of anodic and cathodic currents versus scan rates. **h** Amperometric current-time curves of the WE under various glucose concentrations. **i** Correlation of current and glucose concentration. **j** Performance of the WE in the presence of various interfering chemical species

To investigate the electrical characteristics of the WE, it was evaluated in an electrochemical cell using a standard Ag/AgCl RE and a platinum CE (Fig. 4a). The Bode plots show the carbon and carbon/TTF electrodes have lower impedance while the carbon/TTF/GOx&BSA&GA electrode exhibits dramatically higher impedance across all frequencies (Fig. 4b). Additionally, at a high frequency, the carbon/TTF/GOx&BSA&GA electrode exhibits a larger negative phase angle (Fig. 4c). The Nyquist plot supports the findings above (Fig. 4d), indicating the successful modification of the MN array surface. In operation, when the MN penetrates the skin, the glucose in the ISF will be oxidized by the GOx to form gluconic acid, thereby reducing its cofactor flavin adenine dinucleotide (GOx-FAD) to GOx (FADH₂). The coated TTF reoxidizes the GOx (FADH_2_), generating a current proportional to the glucose concentration and thus achieving glucose detection.

To validate the effectiveness of the MN array WE in glucose sensing, cyclic voltammetry was employed to monitor the oxidation/reduction of glucose (Fig. 4e). The results show that, after the modification with GOx&BSA&GA, the WE exhibits a dramatic increase in current response in the presence of glucose, compared with the carbon and carbon/TTF modification (Supplementary Fig. S8; the TTF modification transformed the carbon electrode from a primarily capacitive system to the one exhibiting faradaic process). For the fully modified electrode, peak currents were measured at various scan rates and were found to increase with the increasing scan rates (Fig. 4f). A clear linear correlation was observed between the redox peak currents and scan rates (Fig. 4g), indicating that the electron transfer between GOx and the electrode primarily followed a diffusion-controlled process^40^. Amperometric responses of the fully modified array were measured at various glucose concentrations (Fig. 4h), revealing a linear range from 2 to 15 mM glucose (Fig. 4i). Additionally, the fully modified electrode demonstrated resistance to interference from a variety of chemical species commonly found in ISF (Fig. 4j). The effects of TTF concentration, GOx concentration, and temperature on amperometric responses were also investigated (Supplementary Fig. S9). Considering both performance and biocompatibility, 10 mM TTF and 20 mg/mL GOx were selected for MN array electrodes in this study.

### In vitro glucose monitoring with the MN array electrodes

**Fig. 5:**
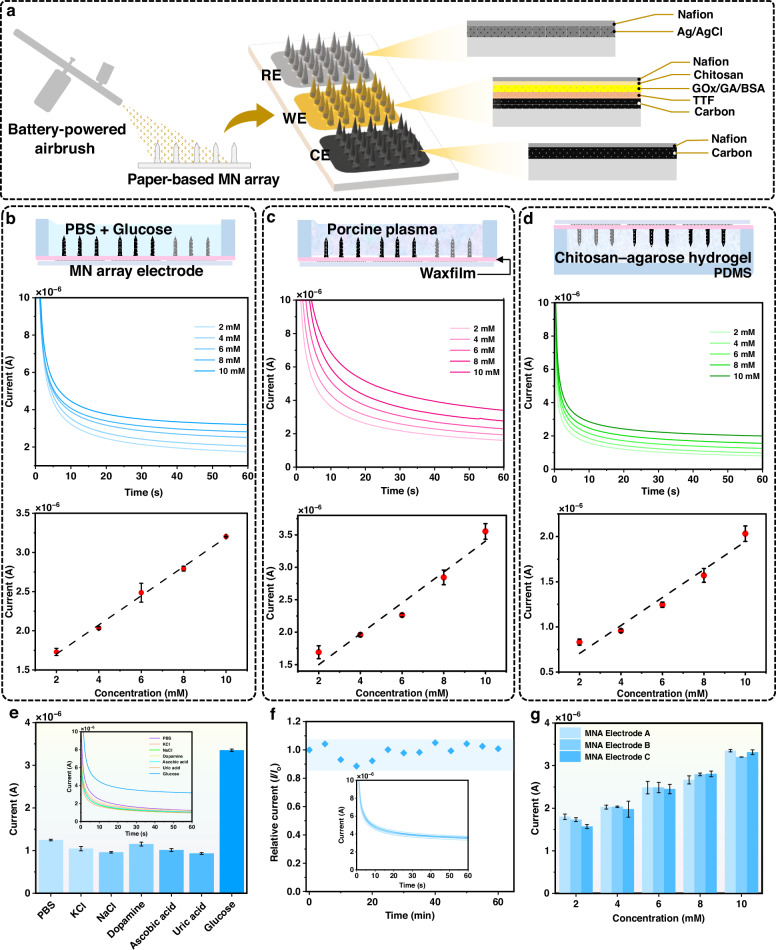
Sensor characterization. **a** Electrodes and their modification components. Subgraphs show the performance of the MN array electrodes in PBS (**b**), porcine plasma (**c**), and chitosan-agarose simulated skin (**d**) at varying glucose concentrations (data collected from three different measurements). **e** Interference study of the MN array electrodes in 0.01 M PBS in the presence of KCl, NaCl, DA, AA, and UA. **f** Stability of the electrodes in PBS containing 10 mM glucose. Twelve repetitive measurements were taken at 5-min intervals over 60 min. **g** Repeatability testing of the biosensors using three MN array electrodes across varying glucose concentrations

To evaluate the performance of the paper-based MN array electrodes (Fig. 5a), glucose concentrations were analyzed in PBS (simulating ISF; Fig. 5b), porcine plasma (simulating human plasma; Fig. 5c), and chitosan-agarose hydrogel (simulating the skin; Fig. 5d). Current values were recorded approximately 60 s after the initiation of amperometry measurement. The results show a linear detection range from 0 to 10 mM for all simulated situations, with sensitivities of 1.85E-7 A/mM (*R*^2^ = 0.995), 2.31E-7 A/mM (*R*^2^ = 0.956), and 1.51E-7 A/mM (*R*^2^ = 0.961), respectively. Additionally, the limits of detection for glucose in PBS, porcine plasma, and chitosan-agarose skin were calculated to be 0.54 mM, 0.743 mM, and 0.686 mM, respectively. While the high sensitivity achieved by our sensor in glucose quantification is comparable to previously reported sensors^16,20,23^ (without WE area normalization), the primary innovation of this device lies in its design and fabrication methodology, which directly addresses key translational barriers outlined in Supplementary Table S2. Under simulated conditions, the paper-based MN array sensor exhibits good selectivity for glucose in the presence of potential interfering chemical species and electroactive molecules (Fig. 5e). Furthermore, the sensor also shows continuous stability (up to four hours) and repeatability (up to 12 measurements within an hour) in glucose biosensing (Fig. 5f and Supplementary Fig. S10), and maintains consistent sensing performance across multiple batches (Fig. 5g).

### Integrated wearable device

**Fig. 6:**
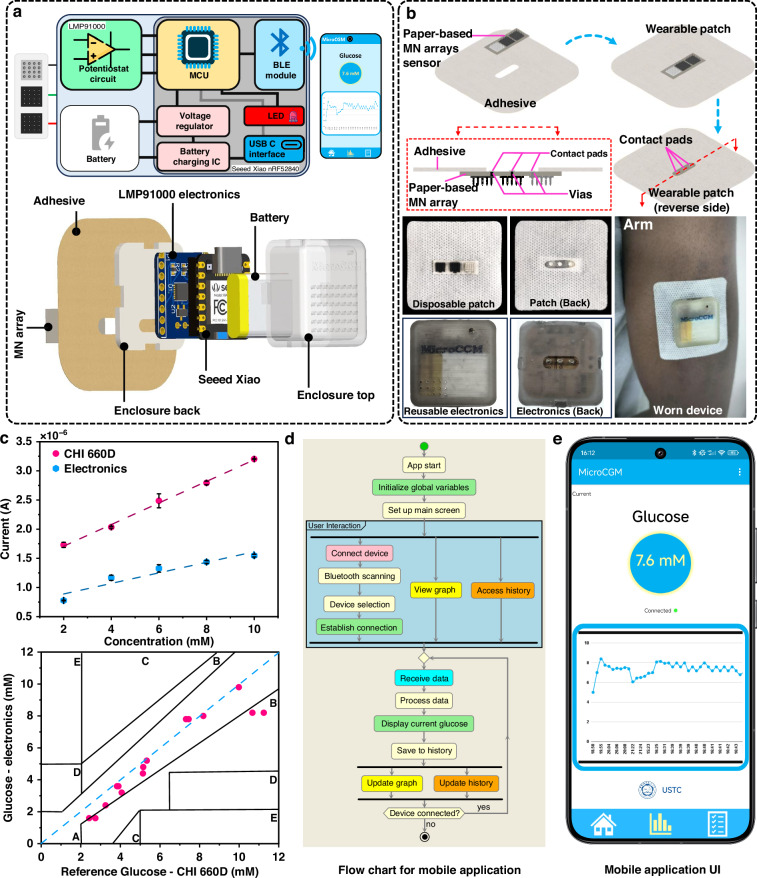
Fully integrated device. **a** Schematic of the fully integrated device, including a disposable flexible MN array patch, a reusable wearable electronic device, and a mobile application. **b** Picture of the disposable flexible MN array patch and the reusable wearable electronic device. **c** Calibration curves measured by the CHI660D electrochemical workstation (pink) and the wearable device designed in this study (blue). **d** Flow chart illustrating the basic functions of the mobile application. **e** User interface of the application

The fully integrated device consists of a disposable flexible MN array electrodes patch, a reusable wearable electronic device for electrochemical measurements and data transfer, and a mobile application (Fig. 6a). The disposable MN array electrode patch comprises paper-based MN array electrodes and a patch fabricated from a breathable medical adhesive (Fig. 6b). The connection between the disposable patch and the electronic device is achieved through 3 pogo pins located on the back of the wearable electronic device and 3 contact pads on the reverse side of the MN array electrodes (these pads are connected to RE, WE, and CE through vias in the paper substrate).

The reusable wearable electronic device consists of a microcontroller development board with Bluetooth functionality (Seeed Xiao BLE Sense) and a potentiostat circuit built around the LMP91000 chip (Supplementary Fig. S11). The Seeed XIAO BLE Sense features a Nordic nRF52840 microcontroller with a 32-bit ARM Cortex-M4 CPU running at 64 MHz and a Floating-Point Unit. Its wireless connectivity (Bluetooth LE 5.2/NFC), low-power features (deep sleep mode), and battery management via the BQ25101 IC make it well-suited for wearable applications. The board is powered by a rechargeable lithium battery (120 mAh), and interfaces with the low-power, I2C-capable LMP91000 analog front-end module for portable electrochemical sensing (in 3-lead amperometric cell mode, the system draws 5–8 mA during active operation, achieving a battery life of approximately 19.5 h). First, the accuracy and reliability of the wearable device were evaluated by comparing its performance with a standard benchtop electrochemical workstation, in terms of Bias voltage stability. The results showed that the wearable device maintained a stable bias voltage of approximately 0.30 V between the WE and RE of the glucose sensor, closely matching the commercial electrochemical workstation’s output. Furthermore, the performance of the wearable device was validated with the MN array electrodes by measuring glucose concentration in porcine plasma. Current responses (i–t curves) and Clarke error grid analysis (Fig. 6c) show that the wearable device’s performance is comparable to that of the CHI660D workstation.

The mobile application functions by wirelessly connecting to the wearable device for data transfer. The user interface allows quick toggling between different views, including connection status, real-time graph, and historical data. Upon startup, the app initializes its core components and presents a user-friendly interface (Fig. 6d, e). Once connected, the app continuously receives glucose data, processes it, and displays the current reading. The real-time graph provides a visual representation of glucose trends over time, enabling users to easily track patterns and fluctuations. Historical data is automatically saved and can be accessed through the history function, offering insights into long-term glucose management (Supplementary Figs. S12 and S13).

### In vivo monitoring of ISF glucose in mice

**Fig. 7:**
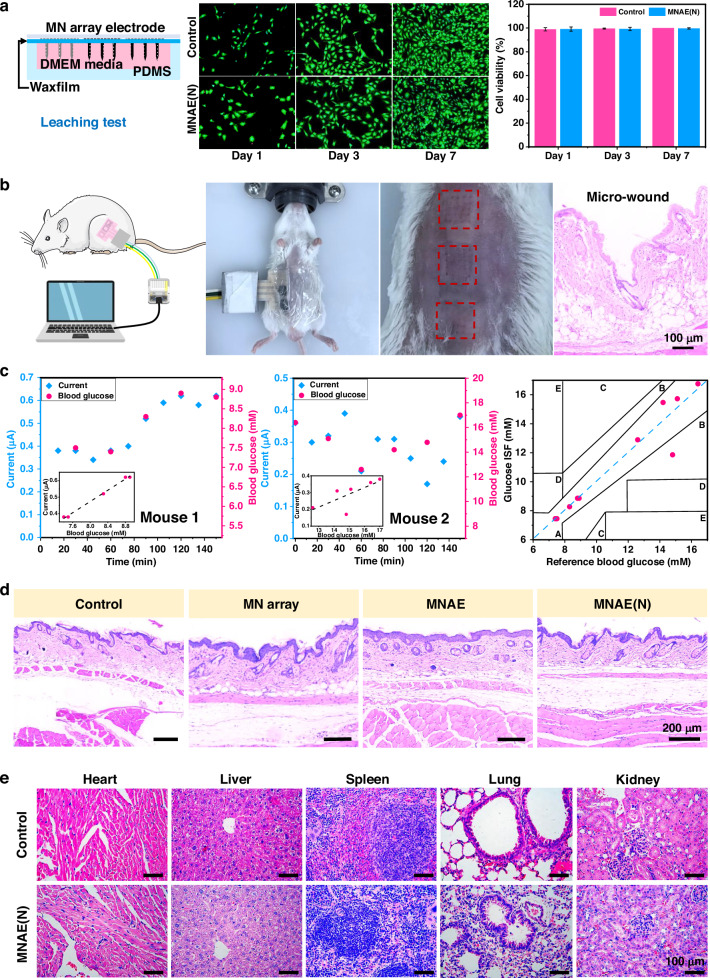
In vivo monitoring of ISF glucose in mice with the MN array patch. **a** Cytotoxicity of the MN array electrodes (MNAE denotes Nafion-uncoated MN array electrodes). **b** Schematic of in vivo monitoring of ISF glucose in mice, images of a mouse with the MN array electrodes inserted and removed, and the HE-stained punctures created by MNs. **c** ISF and blood glucose monitoring for 2.5 h (the results of 2 mice are demonstrated) and a Clark error grid analysis. **d** HE staining analysis of the incised abdominal skin (MNAE(N) denotes Nafion-coated MN array electrodes). **e** HE staining analysis of the major organs after ISF glucose monitoring

Before in vivo monitoring of ISF glucose in mice, the biosafety of the MN array electrodes was evaluated. Cell experiments showed no significant difference in viability between the blank control and MN array groups, confirming the safety of the MN array electrodes (Fig. 7a and Supplementary Fig. S14). When the array electrode was inserted into and removed from the shaved abdomen of mice, the patterned skin structure changes and the punctures created by MNs can be observed (Fig. 7b). However, no visible adverse skin irritation is seen (Supplementary Fig. S15) as the modified TTF hardly dissolves into the skin, suggesting the good biocompatibility of the MN array electrodes. As a proof of concept, the MN array electrodes were tested in vivo. Amperometric measurements were performed at different time points to monitor glucose levels in the ISF of mice (Fig. 7c). Although previous reports have shown a time lag between ISF and blood glucose concentration levels^41,42^. In this study, the time lag was negligible at most time points, with a relative error of less than 1%, demonstrating the excellent glucose-monitoring capability of our MN array electrodes. Further analysis using a Clark error grid showed that nearly all measurements fell in Region A (within 20% of the reference values), confirming that the MN array glucose readings closely matched the reference blood glucose values. In addition, the in vivo glucose-monitoring process did not cause any adverse reactions, inflammatory responses (Fig. 7d), and internal organ damage (Fig. 7e and Supplementary Fig. S16).

## Discussions

This work presents a simple approach for fabricating solid SGR/MCC MN arrays directly on a paper substrate for electrochemical monitoring of ISF glucose. Compared with hollow MN arrays^30,31^, our approach advances existing monitoring technologies by eliminating the need for external pressure, minimizing clogging issues, and reducing sampling delays through the direct placement of electrodes in ISF. In contrast to hollow or porous MN arrays^32–34^, our design enables direct and continuous glucose monitoring. More importantly, the solid MN arrays were fabricated as electrodes directly on a paper substrate rather than on polymer substrates^9,15,16^. This strategy lowers production costs, improves conformity to the body, reduces the risk of skin irritation, and minimizes environmental impact. While paper offers advantages, it also presents main challenges such as ensuring strong MN/substrate adhesion and preventing moisture-induced degradation. Here, we overcame these challenges by implementing a convex fillet base design to distribute stress effectively and by impregnating the substrate with a flexible resin to enhance strength, maintain flexibility, and prevent degradation.

The resin has previously been demonstrated to be safe and stable^43,44^. However, the resin SGR alone was inadequate for producing the desired MNs due to polymerization-induced shrinkage. To address this limitation, a novel composite was formulated by incorporating biocompatible MCC into the UV-curable SGR. Due to the viscous nature of the resulting composite, a custom bi-directional stretching device was designed and fabricated, in conjunction with a stretchable mold, to facilitate the molding of the MN arrays. The biocompatible composite we prepared demonstrates the good properties needed for effective skin penetration, while the stretching device enables precise molding of the viscous substances without the need for vacuum systems or centrifugal forces typically used in conventional fabrication processes. In addition, a simple graphene ink coating method was implemented for electrode functionalization, using a battery-powered airbrush. This eliminates the need for expensive precious metals and specialized equipment reported in previous studies^9,15,16^. Furthermore, the spraying technique produces a uniform coating compared to conventional drop casting.

Currently, most commercial CGM biosensors rely on polymer-based substrates, expensive electrodes, and complex fabrication techniques. In this work, we proposed the use of paper as a substrate for supporting MNs, supplemented by a new MCC/SGR composite for the MN body, a simple fabrication method with a custom-built device for casting the MN array, and a spray coating technique for developing the MN array electrode. This multi-faceted innovation offers a scalable and cost-effective alternative for practical CGM applications.

This study primarily focused on validating the foundational principles and demonstrating the system’s feasibility through laboratory testing, while highlighting the practical value of the low-cost fabrication approach we developed. Although a proof of concept was established in mice, human wearability, long-term stability (e.g., 2 weeks), and error dynamics under diverse conditions remain uninvestigated. Furthermore, the current array-electronics integration and packaging require optimization to improve form factor, usability, and overall user-friendliness. In addition, the sensor may also be further improved through advanced materials or innovative sensing mechanisms to achieve breakthroughs in performance.

## Conclusions

We developed a new disposable paper-based SGR/MCC MN array for continuous ISF glucose monitoring. The MNs were fabricated using a UV-curable SGR/MCC composite, molded with a custom bi-directional stretching device and stretchable mold. The arrays were functionalized as electrodes *via* a simple graphene ink coating process applied with a battery-powered airbrush. The resulting MN array electrodes exhibited good analytical performance, characterized by high sensitivity, selectivity against potential interferents, and robust stability during prolonged use. The potential of this array for minimally invasive glucose monitoring was validated through successful testing in plasma, a hydrogel-based skin mimic, and a mouse model.

## Supplementary information

Supplementary Information

## Data Availability

Data are available from the corresponding author upon reasonable request.
