# Supplementary material for: A wearable paper-based SGR/MCC microneedle array sensor for continuous glucose monitoring
Source: Microsyst Nanoeng. 2026 Jun 3;12:214. doi: 10.1038/s41378-026-01313-1 (PMC13234166; doi:10.1038/s41378-026-01313-1)
Supplement: Supplementary file 1 — Supplementary Information [file 41378_2026_1313_MOESM1_ESM.docx]

**Supplementary Information**

**A Wearable Paper-Based SGR/MCC Microneedle Array Sensor for Continuous Glucose Monitoring**

***Joseph Benjamin Holman^1^,*** ***Talifhani Mushian^2^,*** ***Chen Yang^3^, Yue Jin^3^,*** ***Zhengdi Shi^3^,*** ***Bensheng Qiu^3*^, Chengpan Li^3*^ and Weiping Ding^1*^***

^1^Department of Oncology, The First Affiliated Hospital of USTC, Division of Life Sciences and Medicine, and Department of Electronic Engineering and Information Science, University of Science and Technology of China, Hefei, China

^2^ Hefei National Research Center for Physical Sciences at the Microscale, and Department of Chemistry, University of Science and Technology of China, Hefei, China

^3^Medical Imaging Center, School of Information Science and Technology, University of Science and Technology of China, Hefei, China

*Corresponding authors:

Dr. Weiping Ding: [wpdings@ustc.edu.cn](mailto:wpdings@ustc.edu.cn); ORCID: [0000-0002-3331-1011](http://orcid.org/0000-0002-3331-1011)

Dr. Chengpan Li: licp@ustc.edu.cn; ORCID: 0000-0003-0656-8847

Dr. Bensheng Qiu: [bqiu@ustc.edu.cn](mailto:bqiu@ustc.edu.cn); ORCID: [0000-0003-2987-7378](http://orcid.org/0000-0003-2987-7378)


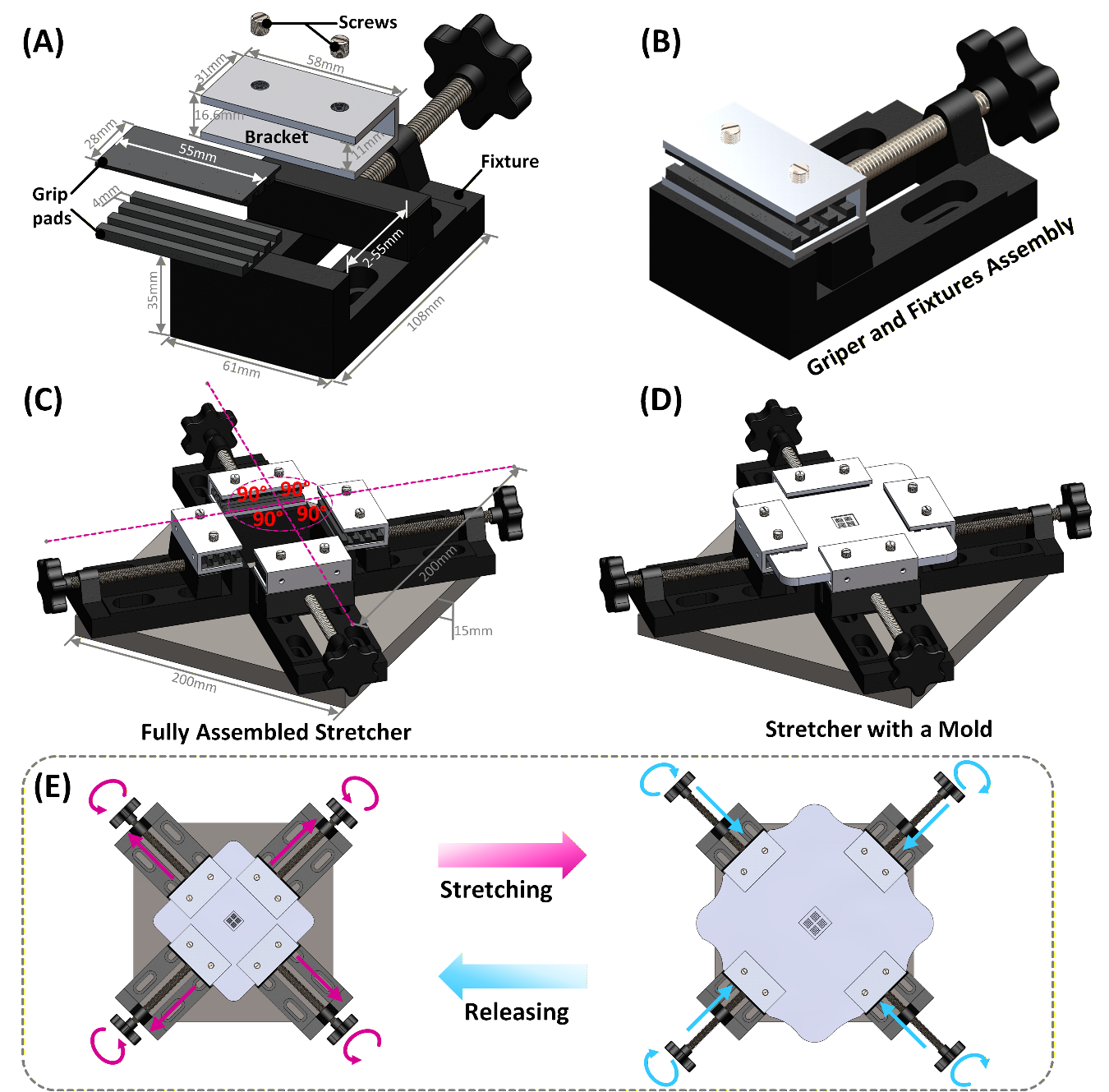


**Figure S1** CAD design of bidirectional stretching device. (A) assembly of components of the bidirectional stretching device (B-D) and (E) the working principles of the stretcher.

**Table S1** Components of the bidirectional stretching device.

| **Part** |  | **Quantity** | **Price (¥)** |
| --- | --- | --- | --- |
| **3D printed grip pad** | 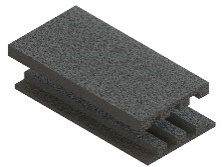 | 4 pairs | / |
| **Aluminum alloy fixing clip bracket** | 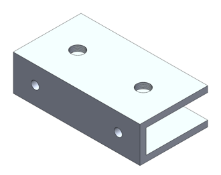 | 4 | ~10 |
| **Multifunctional fixture** | 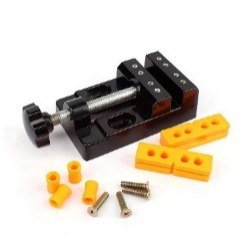 | 4 sets | ~50 |
| **PVC hard board**  (200 × 200 × 15 mm) | **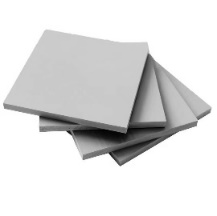** | 1 | ~10 |


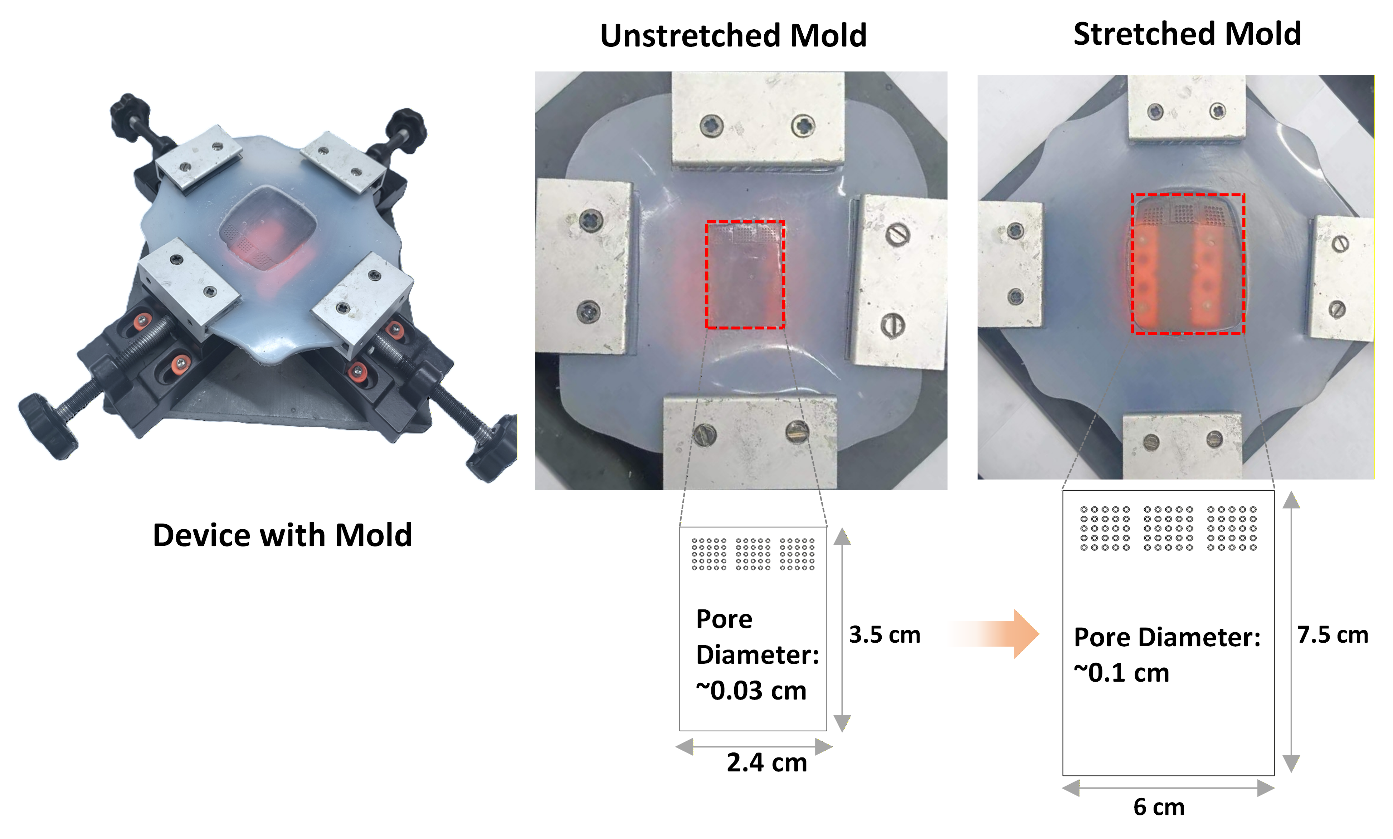


**Figure S2** Stretching the mold with the bidirectional stretching device.

**
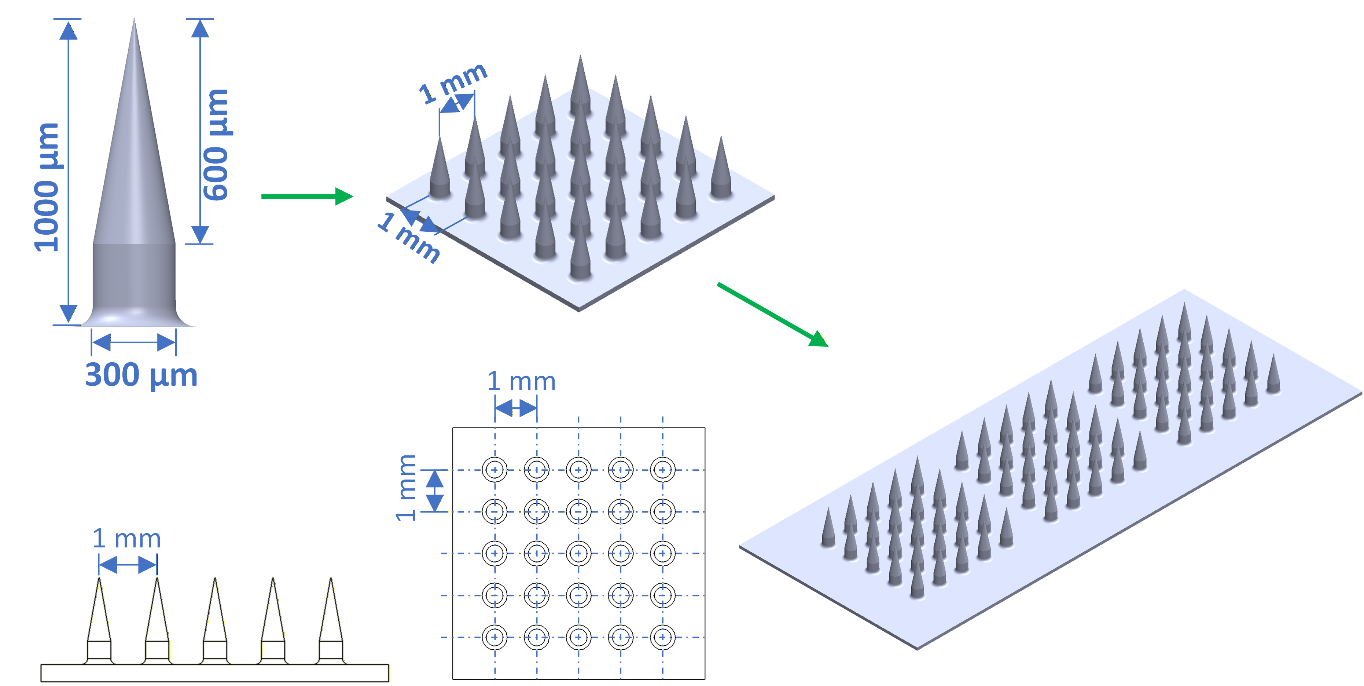
**

**Figure S3** Microneedle design and array dimensions.

**
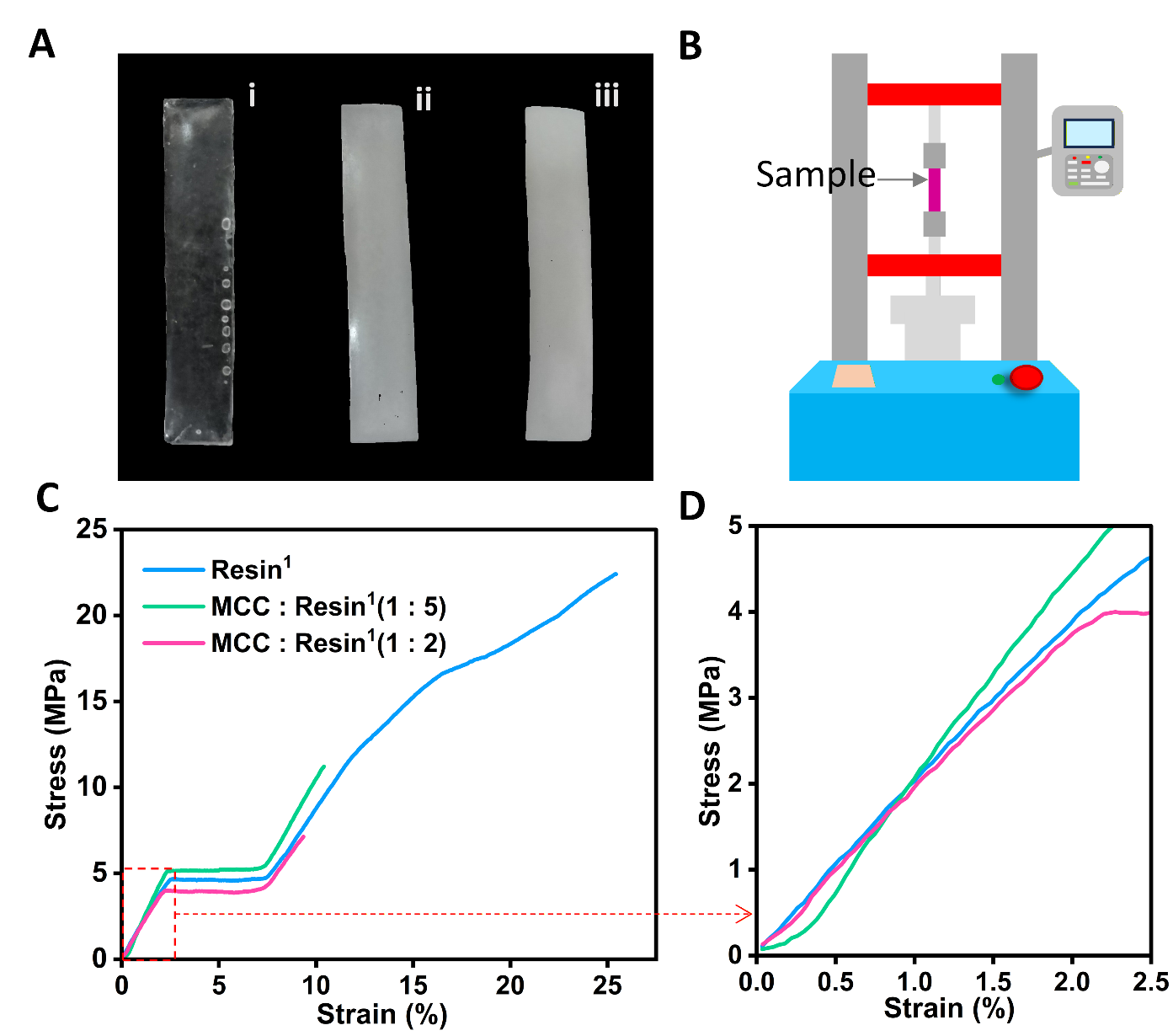
**

**Figure S4** Uniaxial tension test to determine the mechanical properties of the composite. (A) Samples for uniaxial tension test (i. Resin^1^; ii. MCC : Resin^1^ = 1 : 5; ii. MCC : Resin^1^ = 1 : 2). (B) Diagram of the uniaxial tension test. (C) Stress-strain curves of the tested samples. (D) The linear region of the stress-strain curves.


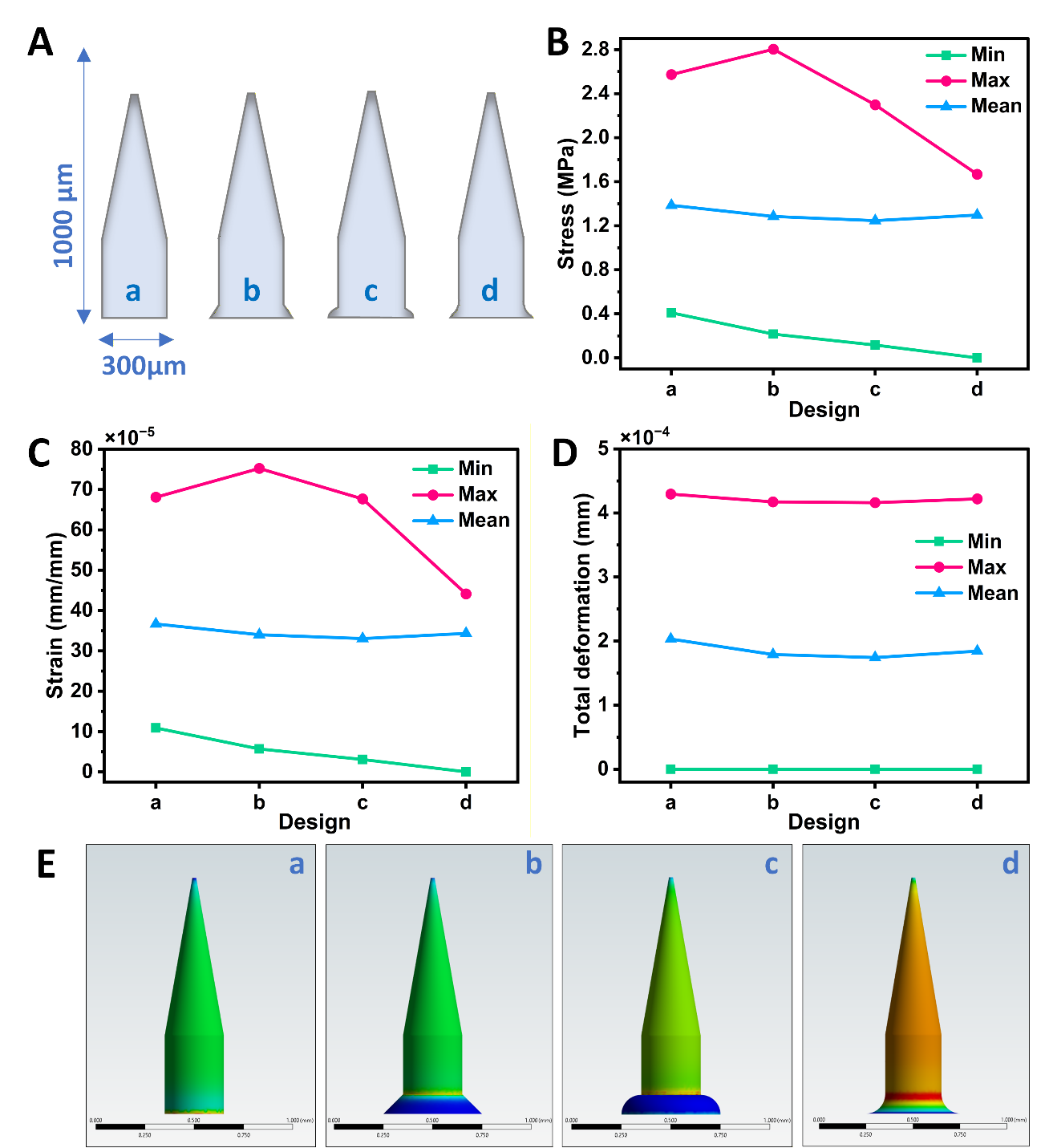


**Figure S5** Finite element analysis of MN base design. (A) MN models (a: straight base; b: mitre fillet; c: convex fillet; and d: concave fillet base joint). Subgraphs (B-E) show stresses, strains, deformations, and stress distribution experienced by the different MN models, respectively.


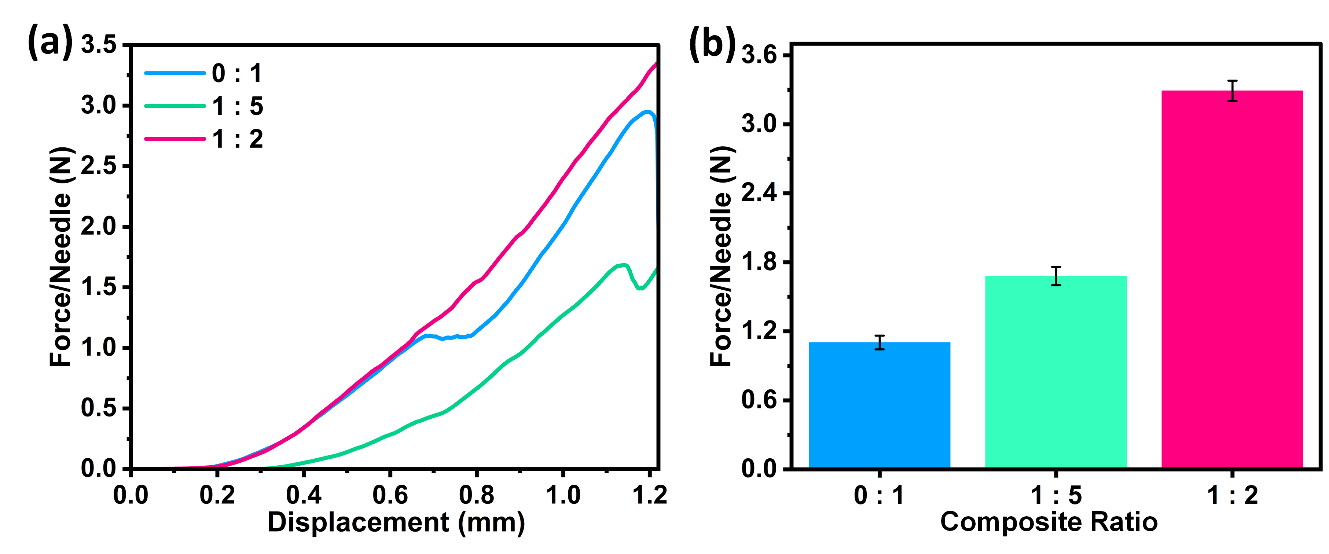


**Figure S6** Mechanical characterization: (A) force per MN vs. displacement and (B) failure force per MN under various MN composition ratios.


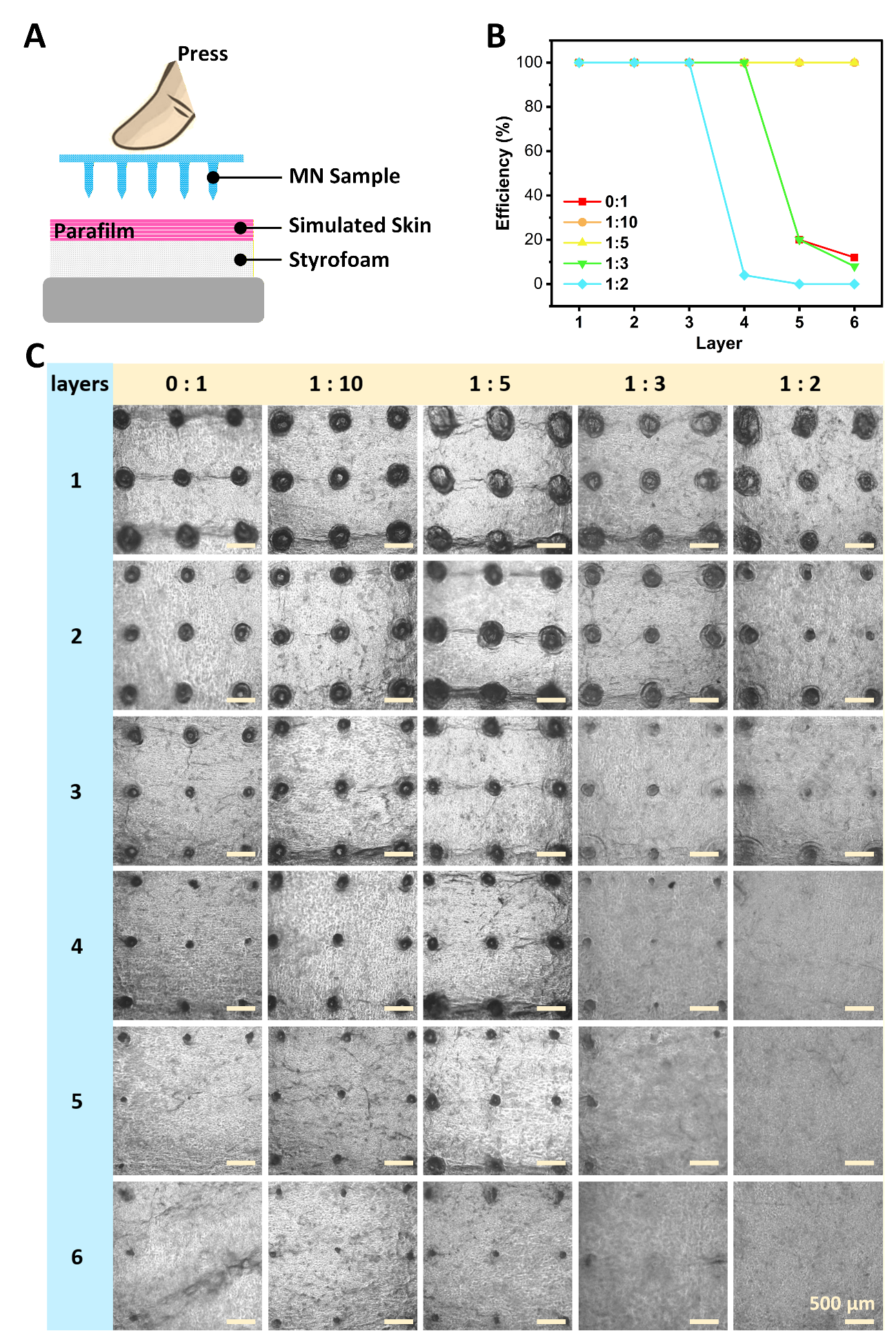


**Figure S7** Skin insertion test. (A) Schematic step-up for the skin insertion test on a Waxfilm-simulated skin. (B) Insertion efficiency of the MN arrays. (C) Penetrated layers.

**
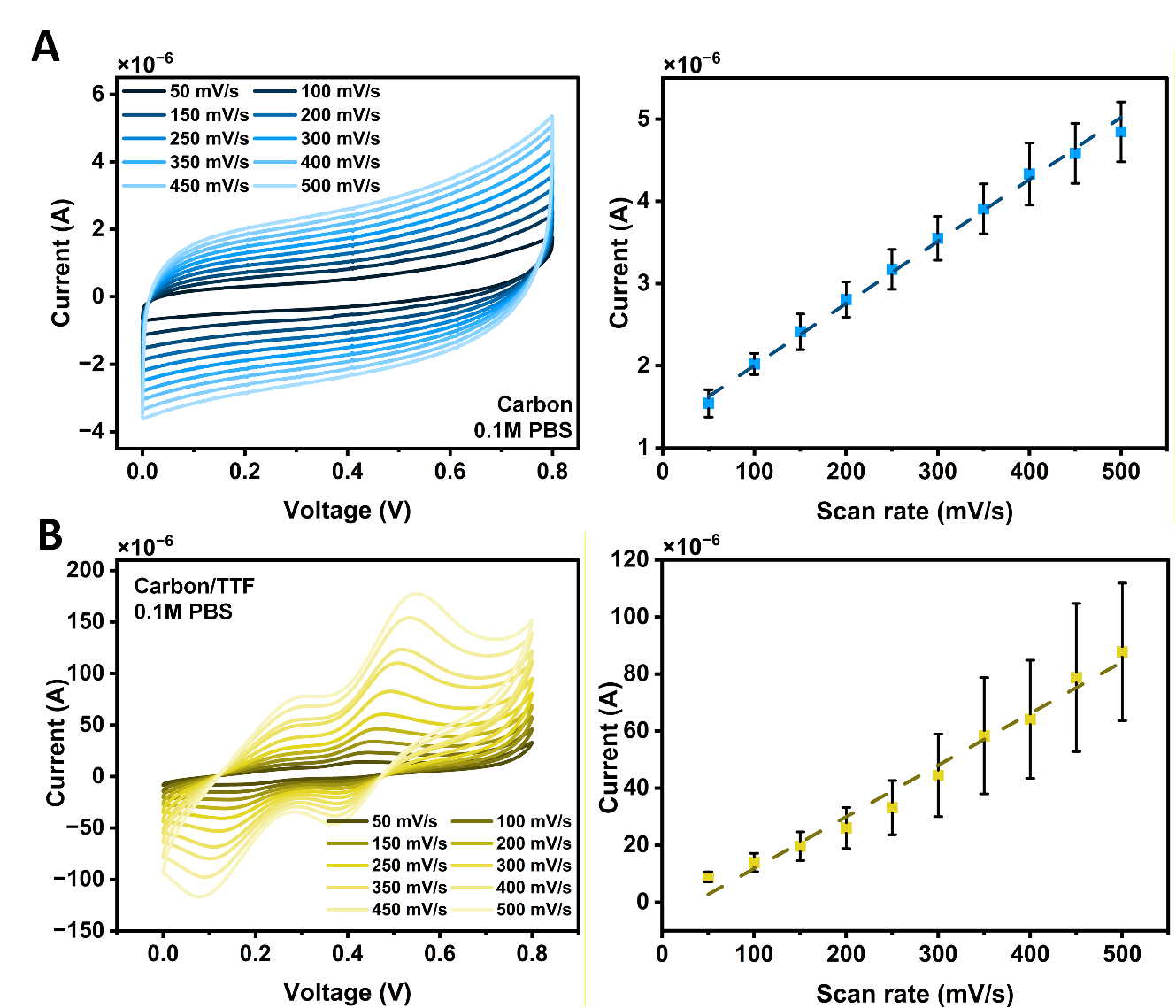
**

**Figure S8** Characterization of MN array electrodes fabricated with (A) carbon and (B) carbon-TTF. In experiments, cyclic voltammograms of electrodes were recorded in 0.01 M PBS using scan rates from 50 - 500 mV/s, and a linear correlation between steady-state current and scan rate was shown for both electrodes.

**
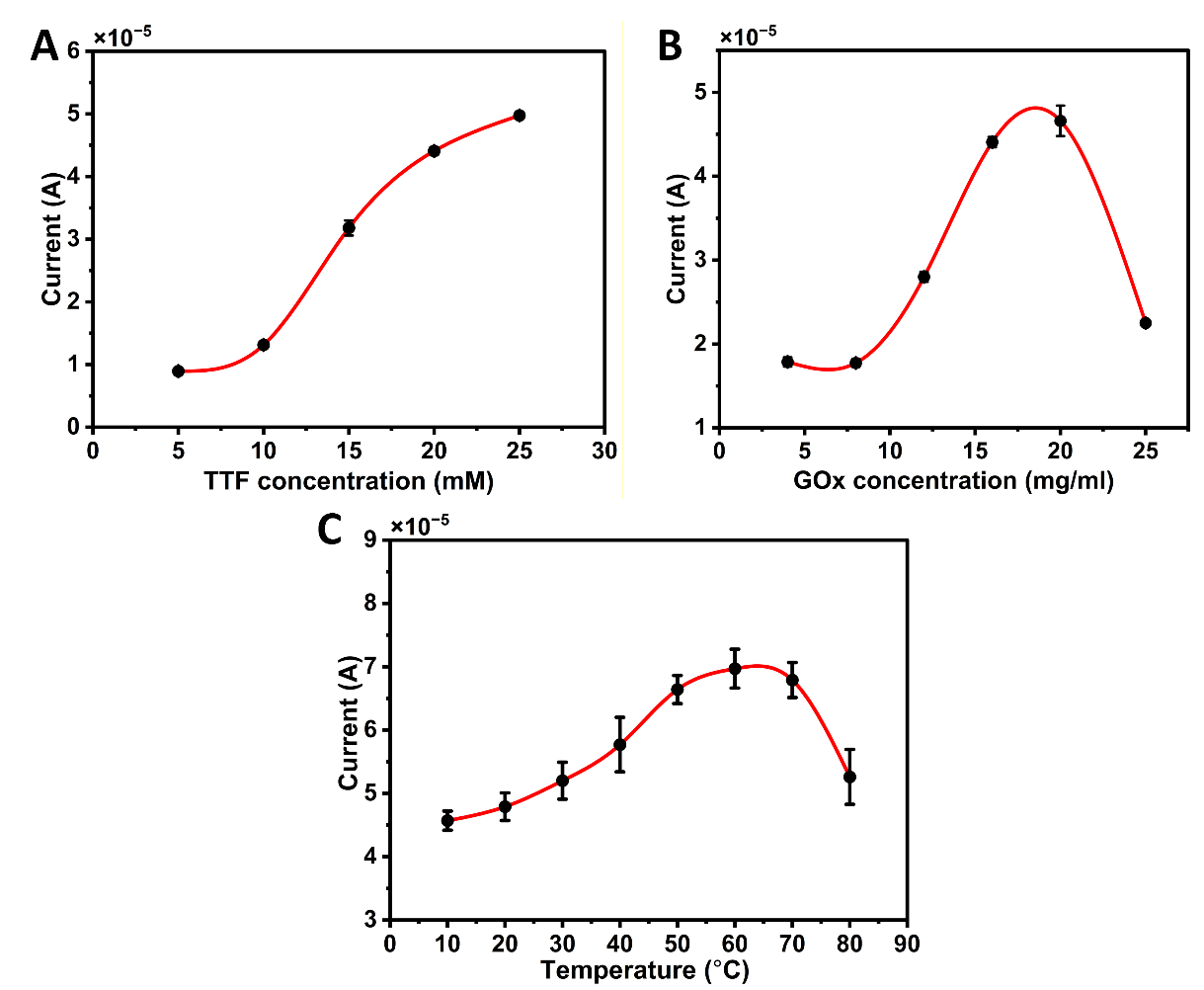
**

**Figure S9** Determination of parameters for the glucose analysis with the proposed working electrode using amperometry. The optimization involved adjusting the concentrations of (A) TTF, (B) glucose oxidase, and (C) temperature in a glucose solution of 5 mM.


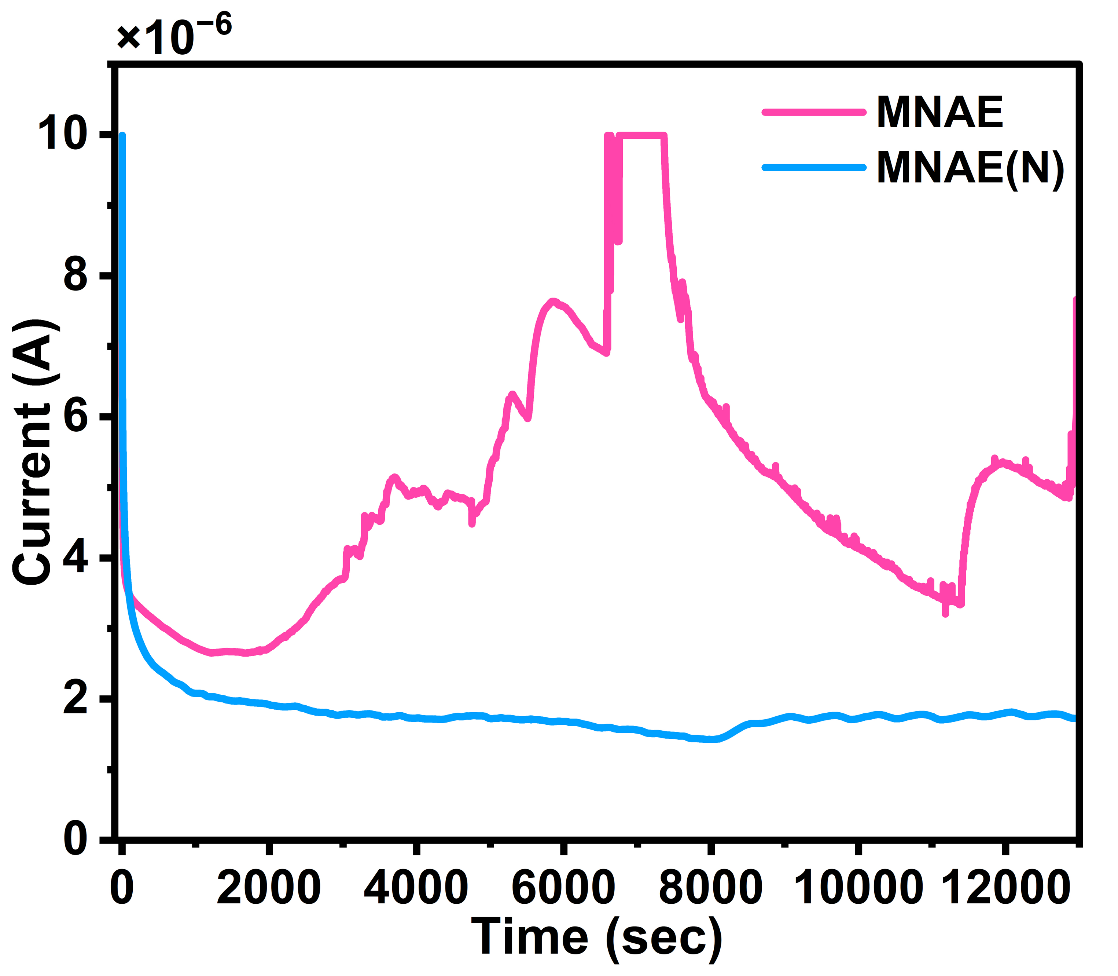


**Figure S10** Stability of the electrodes with and without the Nafion protective layer


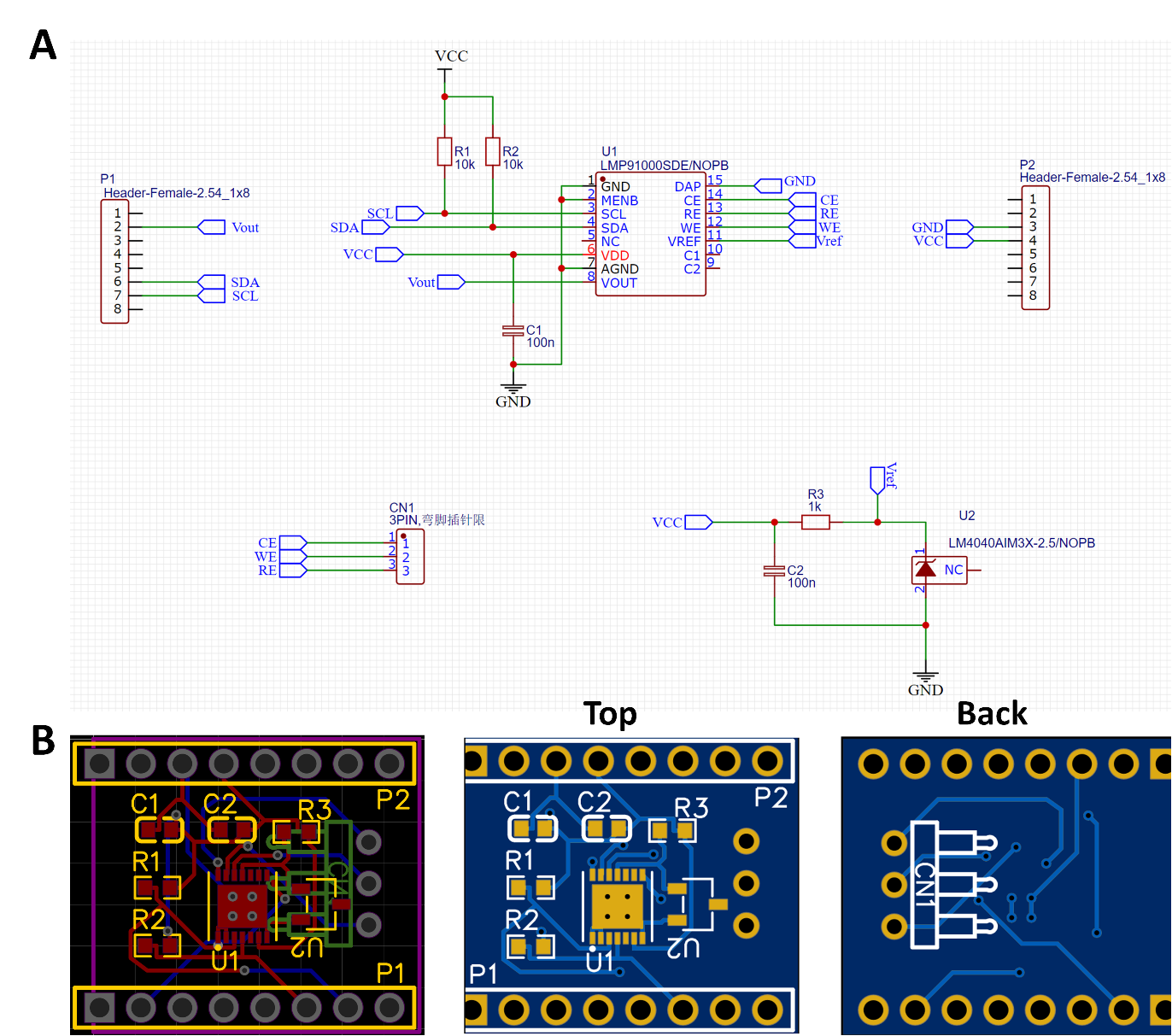


**Figure S11** LMP91000 electronics. (A) Circuit diagram. (B) PCB design of electronics.

**
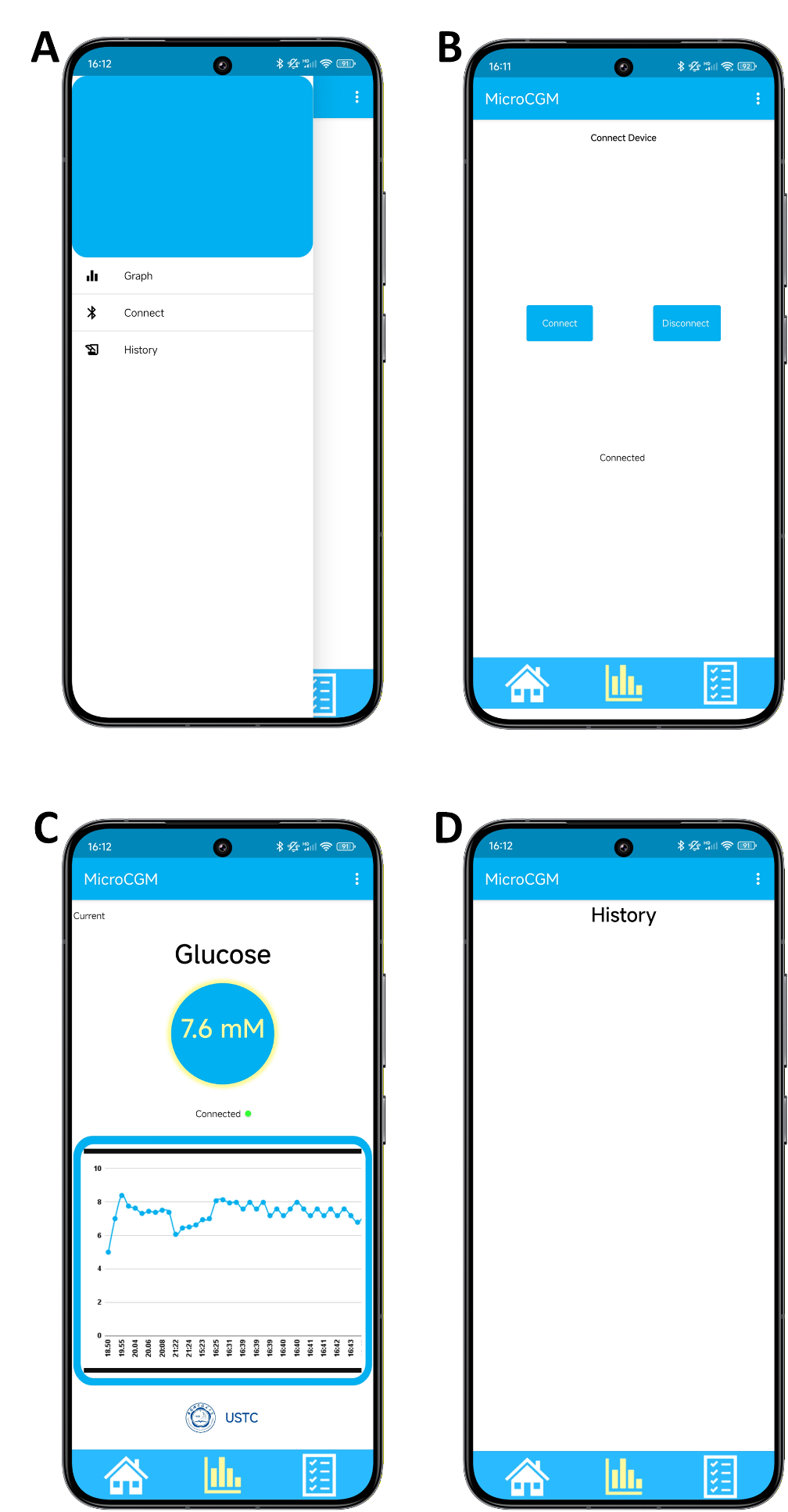
**

**Figure S12** User interface of mobile application: (A) sidebar, (B) connection page, (C) display page, and (D) history page.


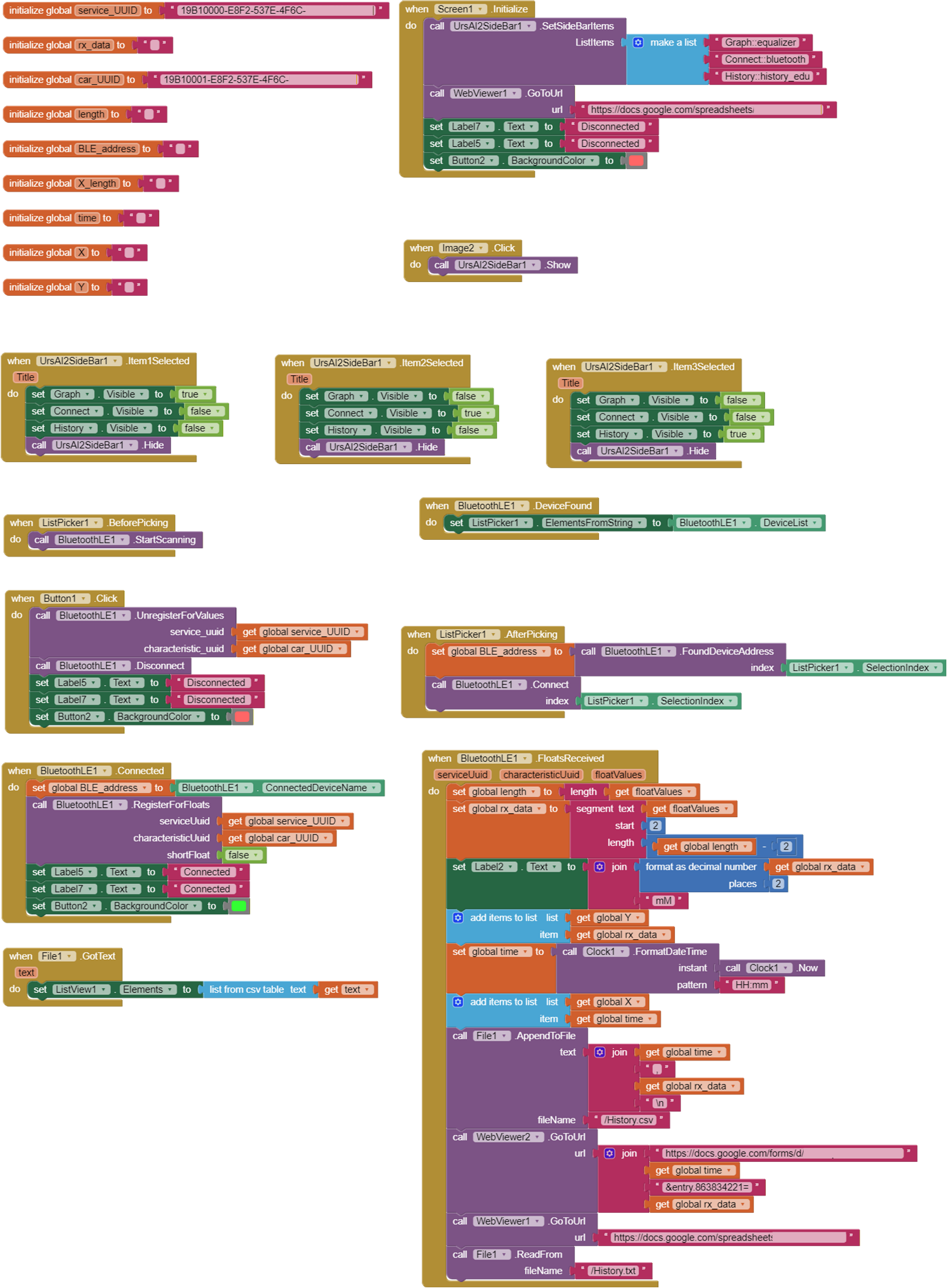


**Figure S13** Code blocks for the mobile application.


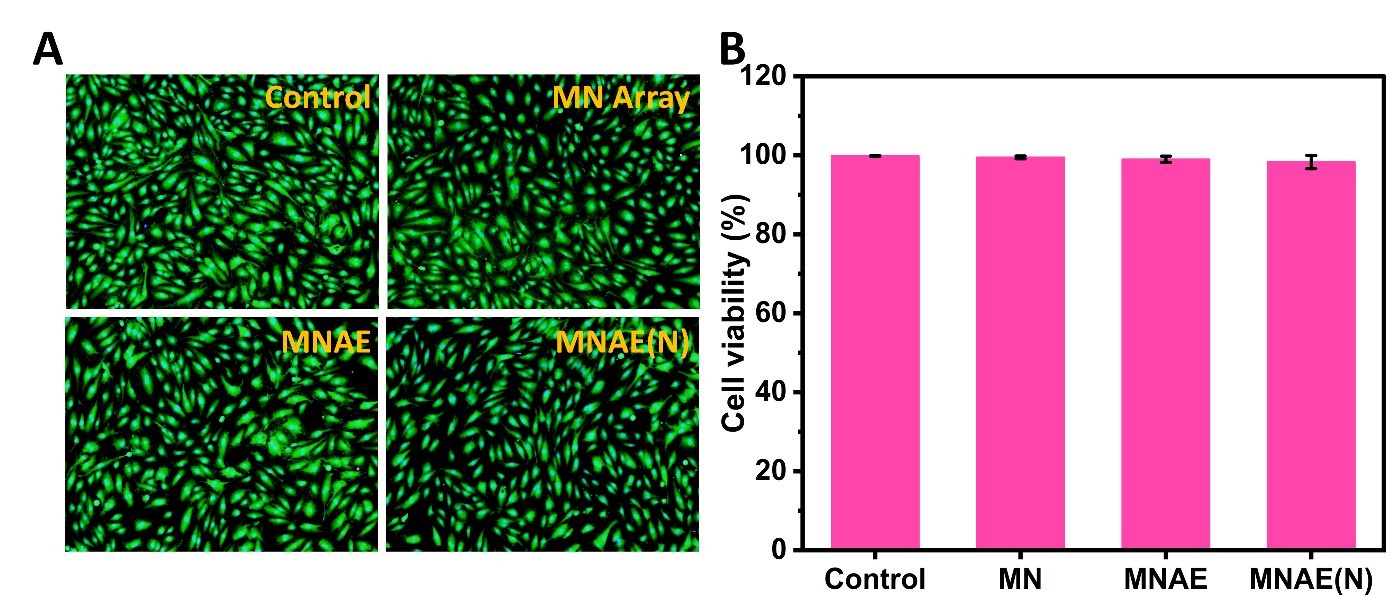


**Figure S14** Cytotoxicity test. (A) Fluorescence images of HUVECs cultured with ECM treated with different MN arrays. (B) Cell viability for different MN arrays (MNAE: Nafion-uncoated MN array electrodes; MNAE(N): Nafion-coated MN array electrodes).

**
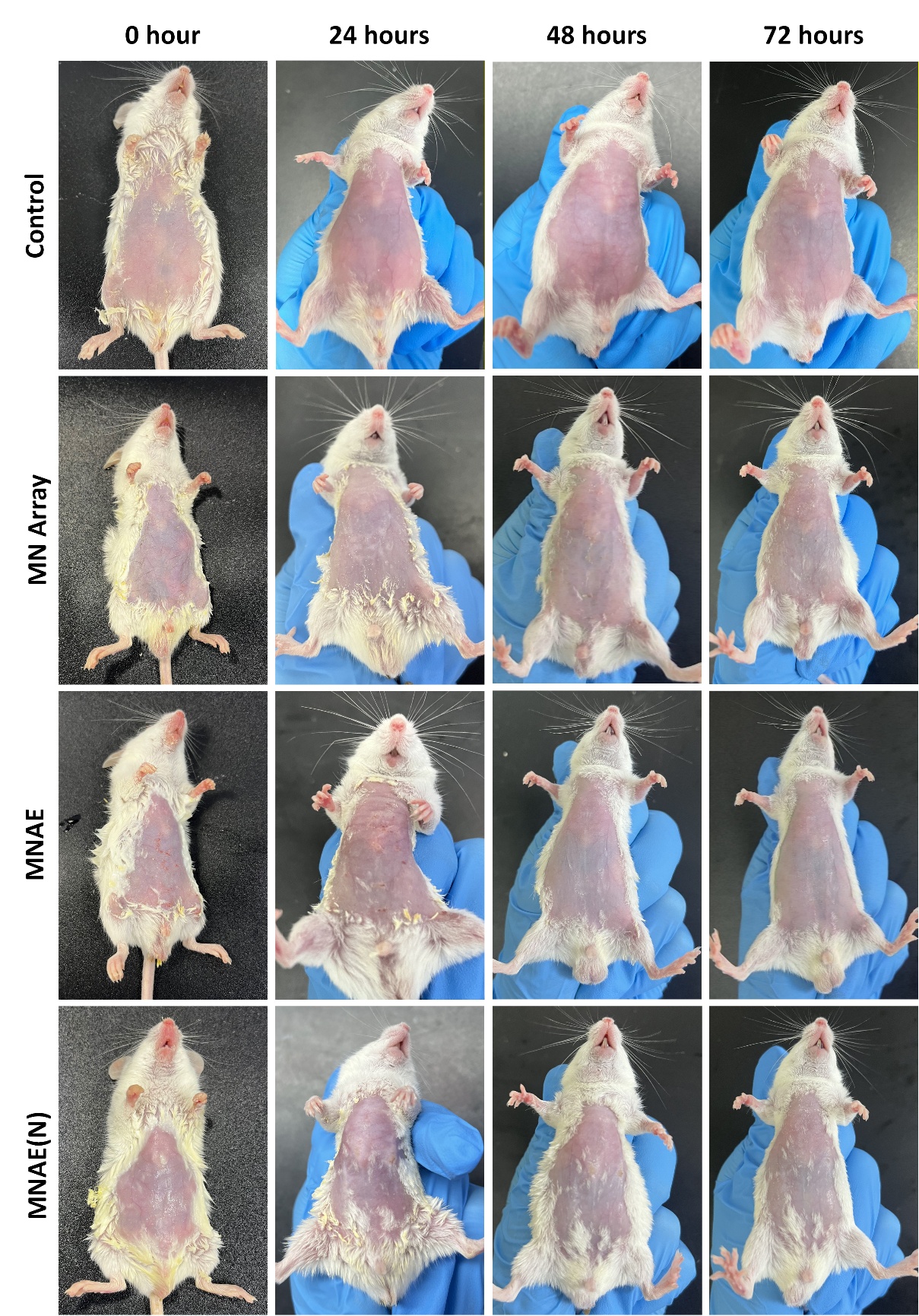
**

**Figure S15** MN array in-vivo dermal irritation experiments (MNAE: Nafion-uncoated MN array electrodes; MNAE(N): Nafion-coated MN array electrodes).

**
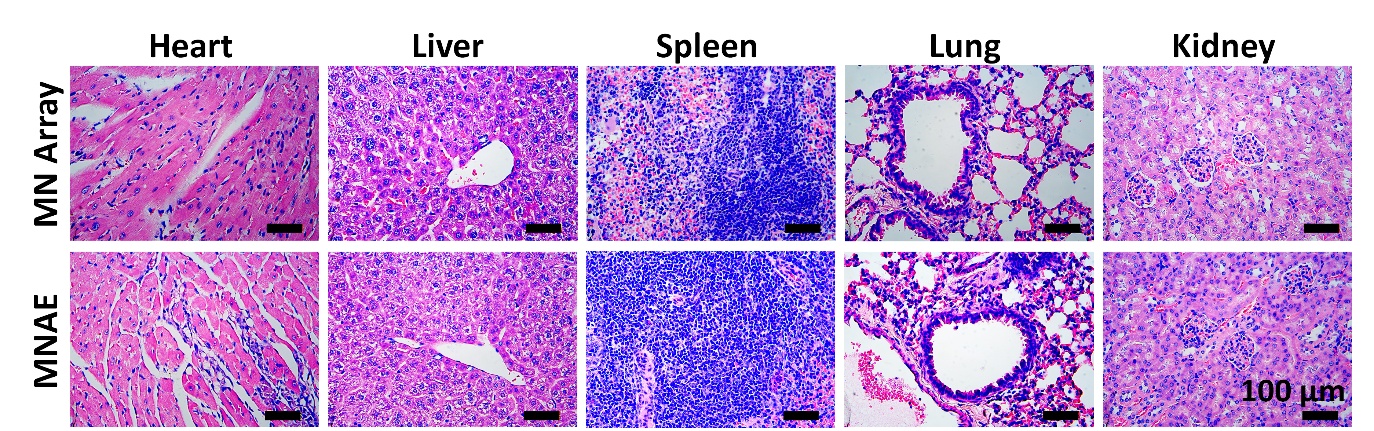
**

**Figure S16** Biosafety of MN array and Nafion-uncoated MN array electrodes (MNAE) *in vivo*. HE staining images of the major organs from mice were obtained.

**Table S2** Comparison between reported MN array devices and this work.

| **Representative**  **Design** | **MN**  **Type** | **MN**  **Materials** | **MN**  **Function** | **MN Base Materials** | **Base Function** | **Fabrication Technique** | **Sensor Type** | **Analyte** | **Requirements** | **Flexible Base** | **Potential Clogging** | **Continuous Monitoring** |
| --- | --- | --- | --- | --- | --- | --- | --- | --- | --- | --- | --- | --- |
| Wu et al. ^1^ | Hollow | PEG-DA | Sampling | PEG-DA Film | Support | Photolithography | Colorimetric | Glucose | Glucose test strips + Camera | No | Yes | No |
| Cheng et al. ^2^ | Hollow | Resin | Sampling | Photocurable Resin | Support | 3D Printing | Colorimetric | Glucose and Lactic Acid | Smart phone | No | Yes | No |
| Liu et al ^3^ | Hollow | Polystyrene | Electrode +  Insulin delivery | Polystyrene | Support | Molding | Electrochemical | Glucose | Electronics + Micropump | No | No | Yes |
| Lee et al. ^4^ | Porous | PDMS | Sampling | PDMS | Colorimetry | Molding | Colorimetric | Glucose | Scanner | Yes | Yes | No |
| Hsieh et al. ^5^ | Porous | PEGDA+  MeHA | Sampling | PEGDA +  MeHA | Support | Molding | Colorimetric | Glucose and Drug | Scanner + Raman spectrometer | No | Yes | No |
| Lee et al. ^6^ | Porous | PLGA | Sampling | Paper | Colorimetry | Hot-Molding | Colorimetric | Glucose | Scanner | Yes | Yes | No |
| Pang et al. ^7^ | Porous | SN+CA | Sampling | Paper | Support | Direct-Drawing | Colorimetric | Glucose, Ca^+^ and Cl^-^ | Centrifuge + Colorimetric kits + Microplate reader | Yes | Yes | No |
| Ghavaminejad et al ^8^ | Porous | DA+HA | Electrode | DA + HA | Support | Molding | Electrochemical | Glucose | Electronics | No | No | Yes |
| Dervisevic et al. ^9^ | Solid | Silicon | Electrode | Silicon | Support | Photolithography and DRIE Etching | Electrochemical | Glucose | Electronics | No | No | Yes |
| Liu et al ^10^ | Solid | Resin | Electrode | Resin | Support | 3D Printing | Electrochemical | Glucose | Electronics | No | No | Yes |
| Tehrani et al. ^11^ | Solid | PMMA | Electrode | PMMA | Support | Micromachining | Electrochemical | Glucose, Lactate and Alcohol | Electronics | No | No | Yes |
| Dervisevic et al. ^12^ | Solid | OrmoStamp | Electrode | OrmoStamp | Support | 3D printing and Soft lithography | Electrochemical | Glucose and Insulin | Electronics | No | No | Yes |
| ***This Work*** | ***Solid*** | ***Resin+MCC*** | ***Electrode*** | ***Paper*** | ***Support*** | ***Stretch-Molding*** | ***Electrochemical*** | ***Glucose*** | ***Electronics*** | ***Yes*** | ***No*** | ***Yes*** |

**PEG-DA**: polyethylene glycol diacrylate; **DA/HA:** Dopamine hydrochloride/sodium hyaluronate; **PDMS**: Polydimethylsiloxane; **SN/CA**: Silica nanoparticles/cellulose acetate composite; **PEGDA/ MeHA**: poly(ethylene glycol) diacrylate/methacrylated hyaluronic acid; **PLGA**: Poly(lactic-co-glycolic acid); **Resin/MCC**: Surgical guide Resin/Microcrystalline cellulose.

**References**

1. Wu TW, You XQ, Chen Z. Hollow Microneedles on a Paper Fabricated by Standard Photolithography for the Screening Test of Prediabetes. *Sensors-Basel* **22**, 4253 (2022).

2. Cheng JL, Huang JK, Xiang Q, Dong HF. Hollow microneedle microfluidic paper-based chip for biomolecules rapid sampling and detection in interstitial fluid. *Anal Chim Acta* **1255**, 341101 (2023).

3. Liu YQ, Yang L, Cui Y. A wearable, rapidly manufacturable, stability-enhancing microneedle patch for closed-loop diabetes management. *Microsyst Nanoeng* **10**, 112 (2024).

4. Lee H, Takeuchi K, Sasaki Y, Takama N, Minami T, Kim B. Porous Microneedle Integrated in Paper based Glucose Sensor for Fluid Channel Interface. In: *2019 IEEE Cpmt Symposium Japan (ICSJ)*) (2019).

5. Hsieh YC*, et al.* Controllable-Swelling Microneedle-Assisted Ultrasensitive Paper Sensing Platforms for Personal Health Monitoring. *Adv Healthc Mater* **12**, 2300321 (2023).

6. Lee H, Bonfante G, Sasaki Y, Takama N, Minami T, Kim B. Porous microneedles on a paper for screening test of prediabetes. *Medical Device & Sensors* **3**, e10109 (2020).

7. Pang YS*, et al.* Porous Microneedles Through Direct Ink Drawing with Nanocomposite Inks for Transdermal Collection of Interstitial Fluid. *Small* **20**, 2305838 (2024).

8. GhavamiNejad P, GhavamiNejad A, Zheng HJ, Dhingra K, Samarikhalaj M, Poudineh M. A Conductive Hydrogel Microneedle-Based Assay Integrating PEDOT:PSS and Ag-Pt Nanoparticles for Real-Time, Enzyme-Less, and Electrochemical Sensing of Glucose. *Adv Healthc Mater* **12**, 2202362 (2023).

9. Dervisevic M*, et al.* Transdermal Electrochemical Monitoring of Glucose via High-Density Silicon Microneedle Array Patch. *Adv Funct Mater* **32**, 2009850 (2022).

10. Liu YQ, Yu Q, Luo XJ, Yang L, Cui Y. Continuous monitoring of diabetes with an integrated microneedle biosensing device through 3D printing. *Microsyst Nanoeng* **7**, 75 (2021).

11. Tehrani F*, et al.* An integrated wearable microneedle array for the continuous monitoring of multiple biomarkers in interstitial fluid. *Nat Biomed Eng* **6**, 1214-1224 (2022).

12. Dervisevic M, Harberts J, Sánchez-Salcedo R, Voelcker NH. 3D Polymeric Lattice Microstructure-Based Microneedle Array for Transdermal Electrochemical Biosensing. *Adv Mater* **36**, 2412999 (2024).
